# Repeatome landscapes and cytogenetics of hortensias provide a framework to trace *Hydrangea* evolution and domestication

**DOI:** 10.1093/aob/mcae184

**Published:** 2025-01-23

**Authors:** Sara Ishiguro, Shota Taniguchi, Nicola Schmidt, Matthias Jost, Stefan Wanke, Tony Heitkam, Nobuko Ohmido

**Affiliations:** Graduate School of Human Development and Environment, Kobe University, Nada-ku, Kobe, 657-8501, Japan; Graduate School of Human Development and Environment, Kobe University, Nada-ku, Kobe, 657-8501, Japan; Faculty of Biology, Technische Universität Dresden, D-01069 Dresden, Germany; Institute of Biology I, RWTH Aachen University, 52056 Aachen, Germany; Institut für Ökologie, Evolution und Diversität, Goethe-Universität Frankfurt, 60438 Frankfurt am Main, Germany; Departamento de Botánica, Instituto de Biología, Universidad Nacional Autónoma de México, Mexico City, Mexico; Abteilung Botanik und Molekulare Evolutionsforschung, Senckenberg Gesellschaft für Naturforschung, 60325 Frankfurt am Main, Germany; Faculty of Biology, Technische Universität Dresden, D-01069 Dresden, Germany; Institut für Ökologie, Evolution und Diversität, Goethe-Universität Frankfurt, 60438 Frankfurt am Main, Germany; Departamento de Botánica, Instituto de Biología, Universidad Nacional Autónoma de México, Mexico City, Mexico; Abteilung Botanik und Molekulare Evolutionsforschung, Senckenberg Gesellschaft für Naturforschung, 60325 Frankfurt am Main, Germany; Faculty of Biology, Technische Universität Dresden, D-01069 Dresden, Germany; Institute of Biology I, RWTH Aachen University, 52056 Aachen, Germany; Graduate School of Human Development and Environment, Kobe University, Nada-ku, Kobe, 657-8501, Japan

**Keywords:** *Hydrangea*, hortensias, repetitive DNA, satellite DNA, cytogenetic, fluorescence *in situ* hybridization, plastome phylogeny

## Abstract

**Background and Aims:**

Ornamental hortensias are bred from a reservoir of over 200 species in the genus *Hydrangea* s.l. (Hydrangeaceae), and are valued in gardens, households and landscapes across the globe. The phenotypic diversity of hortensia cultivars, hybrids and wild relatives is mirrored by their genomic variation, with differences in genome size, base chromosome numbers and ploidy level. We aim to understand the genomic and chromosomal basis of hortensia genome variation. Therefore, we analysed six hortensias with different origins and chromosomal setups for repeatome divergence, the genome fraction with the highest sequence turnover. This holds information from the hortensias’ evolutionary paths and can guide breeding initiatives.

**Methods:**

We compiled a hortensia genotype panel representing members of the sections *Macrophyllae*, *Hydrangea*, *Asperae* and *Heteromallae* and reconstructed a plastome-based phylogenetic hypothesis as the evolutionary basis for all our analyses. We comprehensively characterized the repeatomes by whole-genome sequencing and comparative repeat clustering. Major tandem repeats were localized by multicolour FISH.

**Key Results:**

The *Hydrangea* species show differing repeat profiles reflecting their separation into the two major *Hydrangea* clades: diploid *Hydrangea* species from Japan show a conserved repeat profile, distinguishing them from Japanese polyploids as well as Chinese and American hortensias. These results are in line with plastome-based phylogenies. The presence of specific repeats indicates that *H. paniculata* was not polyploidized directly from the common ancestor of Japanese *Hydrangea* species, but evolved from a distinct progenitor. Major satellite DNAs were detected over all *H. macrophylla* chromosomes.

**Conclusions:**

Repeat composition among the *Hydrangea* species varies in congruence with their origins and phylogeny. Identified species-specific satDNAs may be used as cytogenetic markers to identify *Hydrangea* species and cultivars, and to infer parental species of old *Hydrangea* varieties. This repeatome and cytogenetics information helps to expand the genetic toolbox for tracing hortensia evolution and guiding future hortensia breeding.

## INTRODUCTION

Hortensias are valued for their ornamental flowers (variable in colour, size and shape) as well as their growth form (from shrub to climber), but the underlying genomic and chromosomal variation is still not understood. Yet this is needed to guide the many hortensia breeding initiatives around the globe (Wu and Alexander, 2020). The genus *Hydrangea* s.l. (Hydrangeaceae), which contains more than 200 species and numerous subspecies (De Smet *et al*., 2015), is a group of woody flowering plants, likely originating from North America and East Asia (POWO, 2023; Raman *et al.*, 2023). Molecular phylogenetic studies have shown the monophyly of *Hydrangea* s.l. with two main clades called *Hydrangea* clades I and II (Samain *et al.*, 2010; Mendoza *et al.*, 2013, 2014; De Smet *et al.*, 2015; Raman *et al.*, 2023). Several *Hydrangea* species native to Asia, including *H. macrophylla* and *H. serrata*, were brought to Europe about 200 years ago and since have been improved to serve as ornamental plants (Iwatsuki *et al*., 2001; Rinehart *et al*., 2006; Uemachi *et al*., 2014). During hortensia domestication, the genetic diversity of the cultivars decreased and beneficial traits have been lost (Uemachi *et al*., 2014). Therefore, genetic studies are needed to address the hortensia gene flow during speciation and domestication. So far, simple sequence repeat (SSR) markers and single-nucleotide polymorphisms (SNPs) have been used to assess the genetic diversity and relationships of *Hydrangea* species. With these SSR markers, closely related *H. macrophylla* clones could be distinguished (Reed and Rinehart, 2007; Wu *et al*., 2021). Additionally, the double flower loci of *H. macrophylla* could be linked to SNPs (Nashima *et al*., 2021). These examples demonstrate the importance of assessing the genome space and the relationships across hortensia wild relatives to guide local and global breeding programmes.

The genetic landscape of *Hydrangea* is intricately shaped by variations in chromosome numbers and ploidy levels, which exert profound effects on genome size, intraspecific diversity and varietal identification. Notably, within the genus *Hydrangea*, diploids and polyploids can be present in the same species (e.g. *H. paniculata* individuals with chromosome numbers of 2*n* = 2*x* = 36, 3*x* = 54, 4*x* = 72 and 6*x* = 108; Funamoto and Tanaka, 1988). Furthermore, diploid *Hydrangea* species show variance in their chromosome number with 2*n* = 2*x* = 30–36 chromosomes (Table 1). These differences are due to chromosome rearrangements. Some *Hydrangea* species from Japan and China show dysploidy, e.g. 2*n* = 2*x* = 30 in *H. involucrata* and 2*n* = 2*x* = 34 in different *H. aspera* subspecies (Cerbah *et al.*, 2001). Karyotype variations and genome size differences were not taken into account in the selection process during *Hydrangea* breeding, contributing to difficulties in the identification of the origin of certain *Hydrangea* varieties (e.g. *H. macrophylla* varieties).

**Table 1.** *Hydrangea* genotypes sampled, including (suspected) ploidy level and genome size. In addition, a sixth undesignated genotype (*H.* spec.) was sampled, for which no further information (origin, chromosome number, genome size) is known. Since it is most closely related to *H. macrophylla* and *H. serrata* (see plastome-based phylogeny; Supplementary Data Figs S1 and S2), *H.* spec. is assumed to be diploid as well and its approximate genome size was calculated as mean value from the genome sizes of *H. macrophylla* and *H. serrata*.

Code	Species name/cultivar	Variety	Origin	Ploidy level	Chromosome number (2*n*)	Genome size (Mbp/1C)^1^	Genome size (pg/2C)^1^
Hmacr	*H. macrophylla* Ser. f. *normalis*	Gaku	Japan	Diploid^2^	2*x* = 36^2^	2127	4.34
Hserr	*H. serrata* Ser. var. *yesoensis*	Ezo	Japan	Diploid^2^	2*x* = 36^2^	1660	3.40
Hpani	*H. paniculata* ‘Noriutsugi’	Noriutsugi	Japan	Tetraploid or hexaploid^3^	4*x* = 72 or 6*x* = 108^3^	3484	7.12
Hstri	*H. strigosa*	Strigosa	China	Diploid^2,4^	2*x* = 34^2,4^	1428	2.92
Harbo	*H. arborescens* L. f. *grandiflora* ‘Annabelle’	Annabelle	USA	Diploid^2^	2*x* = 36^2^	770	1.57
Hspec	*H.* spec.	n/a	Unknown	Unknown (diploid)	Unknown	Unknown (1894)	Unknown (3.87)

^1^This work; 1 pg = 978 Mbp.

^2^ Cerbah *et al*., 2001.

^3^Funamoto and Tanaka, 1988

^4^ Mortreau *et al*., 2010.

Chromosomal mapping techniques, including fluorescence *in situ* hybridization (FISH), have the potential to offer this missing information (Heitkam and Garcia, 2023). By localizing individual repetitive sequences along the chromosomes, FISH can shed light on the genomic dynamics underlying chromosome and karyotype evolution (Heslop‐Harrison and Schmidt, 2012; Ohmido *et al.*, 2007; Schmidt *et al*., 2019). For hortensias, cytogenetic methods using ribosomal DNA probes have already provided first insights into karyotype variation and the genomic intricacies among *Hydrangea* species (Van Laere *et al*., 2008; Mortreau *et al*., 2010). To understand the genetic basis of the chromosomal variation and to develop probes for FISH-based cytogenetics, insights into the repetitive genome fraction of hortensias are needed.

Repetitive DNAs (repeats) build the structural backbone of the chromosomes and are the fastest-evolving sequences in a genome. Their fast sequence turnover and their genome-wide amplification and loss can drastically affect karyotypes and overall genome composition. The consequent genetic diversity potentially helps species adapt to environmental changes, thus leading to speciation (Paço *et al*., 2015). Thus, repeats often have species-specific genomic profiles. This helps to understand the relationships of species (Zagorski *et al*., 2020; Kuo *et al.*, 2021) and to support taxonomic efforts (Reed and Rinehart, 2007, 2009; Chae *et al*., 2014; Dodsworth *et al*., 2015; Vitales *et al*., 2020). Depending on the repeat type, their analysis yields different information. Tandem repeats, such as satellite DNAs (satDNAs) and ribosomal DNAs (rDNAs), are often located at key chromosome positions including the (peri)centromeres and (sub)telomeres, as well as specific loci such as the ribosomal RNA genes, which often correspond to secondary constrictions when active, and large chromatin domains like constitutive heterochromatin (Biscotti *et al.*, 2015a, b). These key chromosome positions make tandem repeats the prime choice to develop cytogenetic probes. In contrast, transposable elements (TEs) are usually dispersed throughout the genome (Kubis *et al*., 1998; Sharma and Raina, 2005; Jurka *et al*., 2007). The individual abundances and similarity profiles of the different TE classes, lineages and families provide a fine-grained record of the evolutionary past of an organism (Heitkam *et al*., 2021; Schmidt *et al*., 2023).

To understand the hallmarks of *Hydrangea* genomes and to develop a framework for understanding the history of *Hydrangea* evolution and domestication, we here develop a six-genotype panel for *Hydrangea* (cyto)genomics. This panel encompasses *Hydrangea* species from different geographic regions and reflects the genus-typical ploidy and base chromosome number variation. Here, we focus on *H. macrophylla* (Gaku), *H. serrata* (Ezo), which has recently been proposed as the separate species *H. yesoensis* (Murakami *et al*., 2024), *H. paniculata* (Noriutsugi), *H. strigosa* (Strigosa), and *H. arborescens* (Annabelle). To assess their suitability to serve as a genotype panel for *Hydrangea* genomics, we determined their genome sizes in addition to previously published chromosome numbers and ploidies, and assigned an undesignated genotype (*H.* spec.) within this panel using plastome- and repeatome-based phylogenies. Repeatome analyses using low-coverage genome sequencing data were used to calculate the individual repeat fractions and to harness genomic differences between the genotypes. Finally, analysing tandemly repeated sequences, we developed cytogenetic landmark probes with the aim of tracing karyotype changes.

## MATERIALS AND METHODS

### Plant material and genome size estimation

Genotypes of the genus *Hydrangea* were provided by the Kobe Municipal Arboretum (Kobe, Hyogo, Japan). The analysed genotypes encompass species belonging to both *Hydrangea* clades (I and II) and represent the following sections: *Macrophyllae* (*H. macrophylla* and *H. serrata*), *Hydrangea* (*H. arborescens*), *Asperae* (*H. strigosa*) and *Heteromallae* (*H. paniculata*) following the classification of De Smet *et al.* (2015). Additionally, an undesignated genotype (*H.* spec.) was included to determine its relationships to the above-mentioned species. Further accession details are provided in Table 1.

**Fig. 1.**
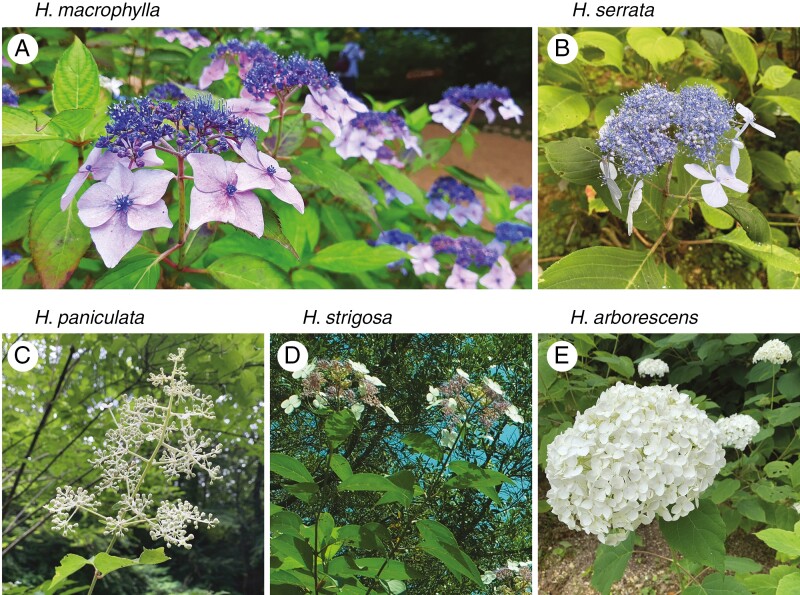
*Hydrangea* species used in this study. (A) *Hydrangea macrophylla* grows in Japan mainland, Honshu (Pacific Ocean side of Kanto district), Izu Islands, Izu Peninsula and Ogasawara Islands. Cutting cultivation is facilitated by its robust properties; leaves are large, thick and glossy. (B) *Hydrangea serrata* grows in all the snowy northern areas of Hokkaido, Honshu (Japan, seaside from Aomori prefecture to Kyoto prefecture) and Kyushu (northern part and Osumi Peninsula). Leaves are rougher and thicker than those of *H. macrophylla*. (C) *Hydrangea paniculata* grows in Hokkaido, Honshu, Shikoku and Kyushu to Yakushima. The plant is the tallest of the Japanese hortensias. (D) *Hydrangea strigosa* is native to central and south-east China. Its leaves are narrower and of a darker green compared with other *Hydrangea* species. (E) *Hydrangea arborescens* is native to the eastern USA. Its leaves are softer than those of the other *Hydrangea* species.

The six *Hydrangea* genotypes analysed in this study represent small deciduous shrubs 2–5 m in diameter. *Hydrangea macrophylla* (‘Gaku-ajisai’ in Japanese; endemic to Japan) grows in Honshu and coastal forests (Iwatsuki *et al*., 2001). The inflorescence is composed of surrounding decorative petal-like-sepals and central non-decorative petals, corresponding to the lacecap inflorescence type (Fig. 1A). Decorative sepals are blue to purple and white and non-decorative petals are blue to purple. *Hydrangea serrata* (‘Ezo-ajisai’ in Japanese; endemic to Japan) grows in northern Japan (Iwatsuki *et al*., 2001). The inflorescence resembles that of *H. macrophylla* (Fig. 1B). *Hydrangea paniculata* (‘Nori-utsugi’ in Japanese) grows all over Japan (Iwatsuki *et al*., 2001). The inflorescence, a long hierarchical structure of white sepals and petals, is distinctly different from that of the two species mentioned above (Fig. 1C). *Hydrangea strigosa* (‘Strigosa’ in Japanese) grows in China. The inflorescence resembles that of *H. macrophylla* (Fig. 1A). However, decorative sepals are white (Fig. 1D). *Hydrangea arborescens* (‘Annabelle’) was propagated in the USA. The inflorescence is composed of only decorative flowers corresponding to the mophead inflorescence type (Fig. 1E).

Flow cytometry was performed for the determination of genome size and ploidy level following Beckman Coulter’s application note with some modifications. Fresh leaf samples were immersed in 1.2 mL of chopping buffer (1.0 % Triton X-100, 140 mm 2-mercaptoethanol, 50 mm NaHSO_3_, 50 mm Tris–HCL, 25 µg mL^−1^ propidium iodide). The leaves were then chopped with a new razor blade. The resulting nuclei suspension was passed through a 20-µm nylon mesh filter and placed on ice, and another 0.6 mL of chopping buffer was added. The nuclei suspension was centrifuged at 97 × g for 4 min. The supernatant was then discarded and the pellet was resolved in another 0.5 mL of chopping buffer. The samples were analysed using a flow cytometer (BD FACSAria™ III Cell Sorter) with measurements for each genotype taken on at least two different days. As an internal standard, *Oryza sativa* subsp. *japonica* ‘Nipponbare’ (433 Mbp/1C; Yu *et al.*, 2005) was used.

### DNA isolation and sequencing

Genomic DNA was isolated from 10–100 mg lyophilized leaf material using the DNeasy Plant Mini Kit (Qiagen, Hilden, Germany). DNA amount and quality were measured with the Qubit™ 4 Fluorometer (Thermo Fisher Scientific, Inc., Waltham, MA, USA), and its integrity was checked via gel electrophoresis. Whole-genome sequencing was performed to generate 2 × 150 bp paired-end (PE) reads with the Novaseq 6000 system (Illumina, San Diego, CA, USA) by Azenta, formerly Genewiz.

### Plastome reconstruction for phylogenetic tree estimation

By sequencing a series of plastid regions and internal transcribed spacer (ITS) regions for an extensive sampling of *Hydrangea* specimens, phylogenetic constructions of the tribe Hydrangeae have been performed (Samain *et al*., 2010; De Smet *et al*., 2015; Raman *et al*., 2023). In this study, we reconstructed the plastid genomes of the newly sequenced genotypes, and used them for phylogenetic tree reconstruction as independent evidence for comparison with ploidy levels and nuclear repeat content as well as positioning the undesignated *Hydrangea* species in the taxonomic framework.

The raw data of the whole sequences were trimmed for adapters and quality with Trimmomatic (v0.39; Bolger *et al*., 2014) using standard settings. The read quality was visualized with FastQC (v0.11.9; Andrews, 2019) before and after trimming. A gold standard for assembly and annotation of high‐quality plastomes based on *de novo* assembly methods and appropriate references for gene annotation was used (Jost and Wanke, 2024). *De novo* plastome assemblies were generated using GetOrganelle (v1.7.7.0; Jin *et al*., 2020), setting the number of rounds to 50. The resulting scaffolds were imported into Geneious (v11.1.5) and finalized to create circular plastomes, where necessary. Correctness of the assemblies was verified via read mappings using BWA (Li and Durbin, 2009). Genes were annotated using the plastome of *Jamesia americana* (NC_044836; Fu *et al*., 2019) as reference. The 5ʹ-end of the *atp*B reference gene was moved to the next canonical in-frame start codon, resulting in a 3-bp shortening of the sequence. The reference for the plastid *ycf*15 gene was taken from the plastome of *Cornus bretschneideri* (MN651479; Li *et al*., 2020), as this gene was not annotated on the *Jamesia* (NC_044836) plastome. TransferRNA boundaries were additionally verified using tRNAscan-SE (Lowe and Chan, 2016). Two different data sets were analysed for their phylogenetic signal, namely the complete plastome (excluding one inverted repeat copy) and the protein-coding and rRNA gene information only. The outgroup sampling comprises representatives from the sister tribe Philadelpheae (*Kirengeshoma palmata* NC_044808; Fu *et al*., 2019) and the sister subfamily Jamesioideae (*Jamesia americana* NC_044836; Fu *et al*., 2019). *Eucnide grandiflora* (NC_044767; Fu *et al*., 2019), a member of sister family Loasaceae, was used for rooting. The alignments were generated using MAFFT (v7.450; Katoh *et al*., 2002; Katoh and Standley, 2013) and manually adjusted in AliView (v1.28; Larsson, 2014). The best-fit nucleotide substitution models for multiple sets of partitions and data were estimated using ModelFinder (Kalyaanamoorthy *et al*., 2017) and used as input for tree reconstruction using both RAxML (allowing only for the GTR model; Stamatakis, 2014) and IQ-TREE (Nguyen *et al*., 2015). Support values are based on 1000 bootstrap replicates. Inferences were then visualized using TreeGraph2 (Stöver and Müller, 2010).

### Read pre-processing and RepeatExplorer2 clustering

After the quality assessment (see section above), raw Illumina reads were trimmed to a final length of 100 nt (20 nt were trimmed from the 5ʹ ends and 30 nt from the 3ʹ ends). Trimmed reads were randomly sampled to obtain 0.1× and 0.05× genome coverage and converted to fasta format using Seqtk (https://github.com/lh3/seqtk). Since no genome size was known for the undesignated genotype *H.* spec., an approximate genome size was assumed based on the plastome-derived phylogeny: *H.* spec. proved to be the closest relative to *H. macrocarpa* and *H. serrata* (Supplementary Data Figs S1 and S2). Thus, the genome size of *H.* spec. was calculated as the mean value of the genome sizes from *H. macrocarpa* and *H. serrata* (1894 Mbp/1C). A graph-based clustering method was performed to identify, characterize and quantify repetitive sequences in each *Hydrangea* genome using the RepeatExplorer2 pipeline on the Galaxy server (https://repeatexplorer-elixir.cerit-sc.cz) with default settings (Novák *et al.*, 2010, 2013). The automatic repeat annotation was manually revised. Repeat abundances were individually assessed based on the read amount within the respective cluster for each genotype (0.1× genome coverage). Additionally, a comparative read clustering was performed with sampled PE reads equivalent to 0.1× or 0.05× genome coverage based on the respective ploidy level (Table 1; *H.* spec. was assumed to be diploid). Since the analysed *H. paniculata* genotype is assumed to be tetraploid (see Discussion section), a 0.05× genome coverage was used to match the genome size of the remaining diploid genotypes (0.1× genome coverage). Each PE read dataset was labelled with a unique five letter code (Table 1). The comparative clustering was performed with default settings.

### Bioinformatic repeat characterization

Candidate species-specific and conserved satDNAs were identified based on the comparative RepeatExplorer2 analysis. A BLAST search of the consensus monomer sequences against *H. macrophylla* long reads was performed to confirm their arrangement in long tandem arrays. Although genomic resources for *Hydrangea* are not abundant, long assembled genome sequences in *H. macrophylla* ‘Aogashima-1’ are available (Nashima *et al*., 2021). Fourteen long-read datasets (DRX222164) were downloaded from the European Nucleotide Archive. Monomeric sequences were aligned to the long reads using BLASTN (Camacho *et al*., 2009), and the long reads containing BLAST hits were assessed with self-dotplots that were constructed using FlexiDot (Seibt *et al*., 2018).

### Probe generation

Amplification of *Hydrangea* satDNA fragments was performed with specific primer pairs designed based on the RepeatExplorer2-derived satDNA monomer consensus sequences (Supplementary Data S1). Detailed information on the primers can be found in Supplementary Data Table S1. The target sequences were amplified using standard PCR reactions with genomic DNA of *H. macrophylla*. An initial denaturation at 94 °C for 3 min was followed by 35 cycles of denaturation at 94 °C for 45 s, primer-specific annealing temperature for 30 s and extension at 72 °C for fragment-specific extension time. A final extension at 72 °C for 5 min was performed. The PCR fragments were purified, cloned and commercially sequenced. Sequenced inserts with the highest identity to the consensus monomer sequences, comprising two or three complete repetitions of the respective monomer, were used as probes for the following hybridization experiments (Supplementary Data S2).

### Chromosome preparation and FISH

Mitotic chromosomes were prepared from actively growing meristems using young buds (bud diameter 0.7–1.7 mm) from *H. macrophylla*. The buds were fixed in methanol:glacial acetic acid (3:1) and stored at −20 °C until required. For sample preparation, a bud was thoroughly washed in water, digested by enzymatic maceration using 2.5 % Pectolyase Y-23 (Seishin Pharmaceutical Co., Ltd, Chiba, Japan) and 1 % Cellulase Onozuka RS (Yakult Honsha Co., Ltd, Tokyo, Japan), and vacuumed for 5 min in a desiccator under −0.1 MPa and incubated at 37 °C for 60 min. The bud was then macerated in a few drops of ethanol:acetic acid (3:1) using fine forceps following the air-drying method (Ohmido *et al*., 1998).

The FISH experiments were performed using the protocol of Schmidt *et al*. (1994) and Liu *et al*. (2021). The probe 18SrRNAgene_Bv_probe1 (Sielemann *et al*., 2023) was used for the detection of the 18S rDNA (labelled with DY415). Specific *Hydrangea* satDNA probes were labelled by PCR with biotin-16-dUTP (Roche Diagnostics) detected by streptavidin-Cy3 (Sigma–Aldrich). Chromosome slides were counterstained with 2 µg mL^−1^ 4ʹ,6-diamidino-2-phenylindole (DAPI). FISH images were photographed directly using an ASI BV300-20A camera coupled to a Zeiss Axioplan 2 imaging microscope with appropriate filters. Image analyses were performed using the Applied Spectral Imaging v3.3 software (ASI).

### Access to data

Illumina whole-genome sequence data are available at EBI under the accession numbers ERR12526782–ERR12526787 (Study ID: ERP151402; Supplementary Data Table S2). The complete plastome sequences of the six *Hydrangea* genotypes analysed in this study have been deposited in GenBank under the accession numbers OR701877–OR701882 (Supplementary Data Table S2). Satellite DNA consensus sequences and the sequences used as FISH probes are available in Supplementary Data S1 and S2. Furthermore, cloned sequences of the satDNAs *Hydrangea*TR01-05a, 07 and 13–15 were submitted to ENA (accession numbers OY742023–OY742031).

## RESULTS

### *A genotype panel to characterize the genus* Hydrangea

The *Hydrangea* genotypes investigated in this study were selected to span remarkable differences in hortensia chromosome numbers, both in terms of base chromosome number and ploidy level (Table 1). Whereas *H. macrophylla*, *H. serrata* and *H. arborescens* represent diploids with 2*n* = 2*x* = 36 chromosomes, the diploid chromosome set of *H. strigosa* comprises only 2*n* = 2*x* = 34 chromosomes (Cerbah *et al.*, 2001). On the other hand, *H. paniculata* was included as a polyploid hortensia plant.

Although more genomic data of hortensias has become available in the last decade, most of the available phylogenies include only a fraction of the large *Hydrangea* diversity. As a result, *Hydrangea* systematics is still in flux. Exploring the possibility of using the repeatome to classify a species without genetic information, one undesignated species from the Kobe Municipal Arboretum (*H.* spec.) was included in our genotype panel. We derived a phylogeny of all genotypes used in this study based on their plastomes (protein-coding and rRNA regions as well as whole plastid genomes; Supplementary Data Figs S1 and S2). Plastome reconstruction resulted in complete, circular genomes for all accessions. The plastid chromosomes possess the typical quadripartite structure and range from 157 617 to 158 055 bp in size (Supplementary Data Fig. S1). The reconstructions share identical gene and intron content. The tree reconstructions (Supplementary Data Fig. S2) depict extremely short branch lengths for *H. macrophylla*, *H. serrata* and *H.* spec., being the likely reason for varying topologies, depending on the data set used (bootstrap support values between 52 and 81; Supplementary Data Fig. S2), implying that *H.* spec. is very closely related to the two members of *Hydrangea* clade II, and is thus also a member of the section *Macrophyllae*.

Similar to ongoing phylogenetic investigations, information on *Hydrangea* genome sizes is patchy and sometimes varies considerably (Plant DNA C-values Database). Therefore, we estimated the genome sizes for the *Hydrangea* genotypes designated as belonging to a certain species (Table 1). The genome sizes among these five *Hydrangea* species range from 770 ± 83 Mbp/1C in *H. arborescens* to 3484 ± 52 Mbp/1C in *H. paniculata*. Among the Japanese diploid *Hydrangea* species, the genome size of *H. macrophylla* (2127 ± 273 Mbp/1C) is larger than that of *H. serrata* (1660 ± 108 Mbp/1C), although their chromosome numbers are the same. In contrast, *H. strigosa* shows both a reduced chromosome number (34 chromosomes) and a lower genome size (1428 ± 94 Mbp/1C) compared with *H. macrophylla* and *H. serrata*. The observed variation in origin, chromosome number and genome size indicates that the *Hydrangea* species of this study represent a powerful genotype panel for the analysis of *Hydrangea* diversity.

### Hydrangea *genomes are mainly composed of Ty3/Gypsy retrotransposons*

**Fig. 2.**
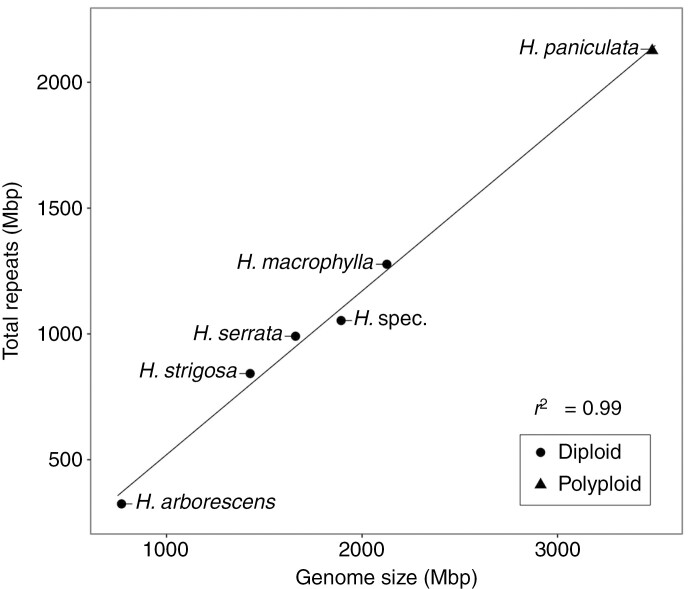
Correlation between genome size and repeat fraction among six different *Hydrangea* genotypes. Symbol shapes indicate different ploidy levels.

To estimate and compare the repeat compositions among the six *Hydrangea* genotypes, clustering analyses were individually performed with the RepeatExplorer2 pipeline. The number of reads per sample was between 0.77 and 3.48 million, corresponding to a 0.1× genome coverage of the respective genotype (Table 1). Based on the threshold of 0.01 % (minimum number of reads within a cluster; at least 71–348 reads), 338–403 clusters were annotated as repetitive DNA, accumulating in 204–260 superclusters. This equals an overall repeat proportion of 42.15–61.08 %, highly correlating with genome size (*r*^2^ = 0.99; Fig. 2; Table 2).

**Table 2.** Genome proportions (%) of different repeat classes among six *Hydrangea* genotypes (codes as in Table 1). The corresponding absolute repeat amounts (Mbp) based on the genome sizes listed in Table 1 are shown in Supplementary Data Table S3.

Repeat		Lineage	Clade	Genome proportion (%)					
				Hmacr	Hserr	Hpani	Hstri	Harbo	Hspec
**LTR retrotransposon**	**Ty1/Copia**	Retrofit/Ale		1.44	1.58	2.43	1.24	1.05	1.67
		Retrofit/Alesia		0.00	0.00	0.00	0.01	0.00	0.00
		Oryco (Ikeros)		0.74	0.66	0.47	0.56	0.35	0.77
		TORK/Tork		0.10	0.12	0.67	0.57	0.14	0.07
		TORK/Angela		4.36	3.84	2.39	0.49	1.15	3.72
		TORK/TAR		0.73	0.54	0.60	1.19	0.33	0.77
		TORK/Ivana		0.00	0.02	0.03	0.17	0.00	0.00
		SIRE		0.64	0.41	0.35	0.36	0.11	0.61
		Bianca		0.07	0.09	0.23	0.36	0.06	0.07
		**Total Ty1/Copia**		**8.08**	**7.28**	**7.17**	**4.95**	**3.19**	**7.68**
	**Ty3/Gypsy**	Chromovirus	CRM	0.60	0.67	0.50	0.53	0.38	0.61
			Tekay	13.29	13.14	27.65	17.61	19.25	11.82
		Non-chromovirus	Athila	8.63	7.85	3.02	1.80	5.92	6.85
			Ogre	13.88	12.84	4.87	16.36	0.53	13.19
			Retand	3.07	3.39	2.90	3.16	0.28	3.10
		**Total Ty3/Gypsy**		**39.47**	**37.89**	**38.94**	**39.47**	**26.36**	**35.57**
	Unclassified LTR			3.91	2.89	2.59	2.89	1.27	0.91
	**Total LTR**			**51.46**	**48.05**	**48.70**	**47.31**	**30.82**	**44.16**
LINE				0.52	0.47	0.70	0.24	0.63	0.44
Pararetrovirus				0.14	0.00	0.19	0.08	0.00	0.00
	**Total retrotransposons**			**52.12**	**48.52**	**49.59**	**47.63**	**31.45**	**44.60**
**DNA transposons**	TIR	EnSpm_CACTA		0.02	0.00	0.11	0.00	0.01	0.02
		hAT		0.09	0.04	0.25	0.04	0.02	0.15
		MuDR_Mutator		0.13	0.06	0.26	0.13	0.04	0.12
		PIF_Harbinger		0.00	0.00	0.09	0.00	0.00	0.00
	Helitron			0.00	0.00	0.02	0.00	0.00	0.00
	**Total DNA transposons**			**0.24**	**0.10**	**0.73**	**0.17**	**0.07**	**0.29**
**Tandem repeats**	satDNA			1.16	2.37	0.57	1.12	0.24	1.31
	rDNA	35S		0.23	0.99	1.35	0.68	0.91	0.41
		5S		0.00	0.10	0.01	0.11	0.03	0.00
Unclassified repeats				6.30	7.62	8.82	9.32	9.44	8.91
**Total repeats**				**60.03**	**59.69**	**61.08**	**59.01**	**42.15**	**55.52**

Bold indicates the types of repeats and their total values.

**Fig. 3.**
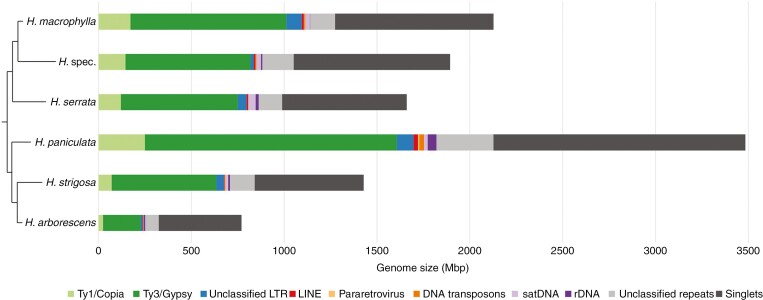
Repeat composition of the six *Hydrangea* genotypes. The cladogram is based on the plastome-derived phylogeny (protein-coding and rRNA regions of the plastid genome; see Supplementary Data Fig. S2). The composition of major repeat classes in absolute values (Mbp) is shown in relation to total genome size. For relative repeat proportions as percentages of total genome size, see Supplementary Data Fig. S3.

**Fig. 4.**
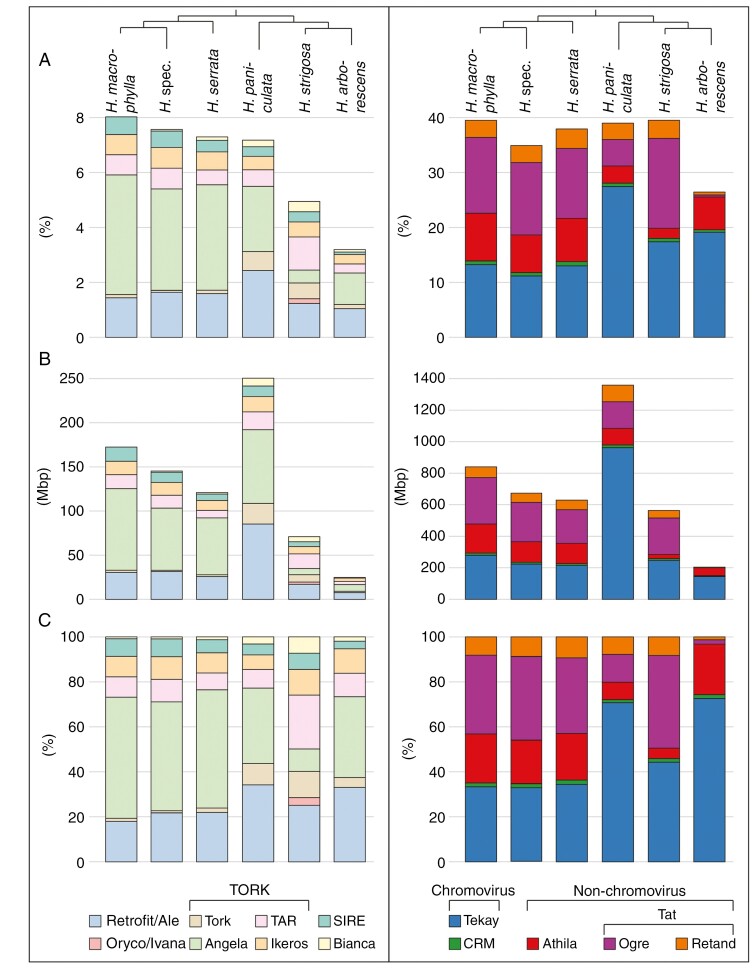
Repeat composition of LTR retrotransposons (left, Ty1/Copia; right, Ty3/Gypsy) among the six *Hydrangea* genotypes. The cladogram is based on the plastome-derived phylogeny (protein-coding and rRNA regions of the plastid genome; see Supplementary Data Fig. S2). (A) Percentage contribution of the respective Ty1/Copia (sub)lineage or Ty3/Gypsy clade to the *Hydrangea* genomes. Note the different scales. (B) Contribution of the respective Ty1/Copia (sub)lineage or Ty3/Gypsy clade to the *Hydrangea* genomes as absolute values (Mbp). Note the different scales. (C) Relative composition of LTR retrotransposon fractions.

The LTR retrotransposons are the most abundant repetitive elements within the *Hydrangea* genomes, accounting for 30.82–51.46 % (Fig. 3; Table 2; Supplementary Data Fig. S3). Retrotransposons from the Ty3/Gypsy superfamily are the predominant elements within this fraction (26.36–39.47 %), represented by four clades: Tekay and CRM from the chromovirus lineage, and Athila and Tat (subclades Ogre and Retand) from the non-chromovirus lineage (Fig. 4). Tekay elements stand out, showing genome proportions of 11.82–27.65 %. Ogre elements show the highest variety in abundance, with only 0.53 % in *H. arborescens* compared with 16.36 % in *H. strigosa* (Table 2). However, Athila elements also show variance in abundance as they occupy 5.92–8.63 % of the genomes from *H. macrophylla*, *H. serrata*, *H. arborescens* and *H.* spec., whereas they only account for 1.80 and 3.02 % in *H. strigosa* and *H. paniculata*, respectively. Retand elements show a species-specific low abundance in *H. arborescens* with a genome proportion that is less than one-tenth of that of the other *Hydrangea* species (Fig. 4).

Ty1/Copia retrotransposons account for 3.19–8.08 % of the *Hydrangea* genomes (Fig. 3; Table 2; Supplementary Data Fig. S3), being composed of five lineages: Retrofit (clades Ale and Alesia), Oryco/Ivana, TORK (clades Tork, Angela, TAR and Ikeros), Bianca and SIRE (Fig. 4). Among diploid *Hydrangea* species with Japanese origin (*H. macrophylla* and *H. serrata*), Angela elements are predominant, occupying 3.84–4.36 % of the respective genome. On the other hand, Angela and Ale elements are similarly abundant in the genomes of *H. paniculata* (~2.5 %, each) and *H. arborescens* (1 %, each), whereas Ale elements are the most abundant Ty1/Copia retrotransposons in *H. strigosa* (1.24 % of the genome; Table 2). DNA transposons contribute 0.07–0.73 % to the *Hydrangea* genomes (Table 2). In regard to tandem repeats, satDNAs occupy 0.24–2.37 % of the respective genome.

### *Repeat composition patterns distinguish Chinese and American from Japanese* Hydrangea *species*

To compare the repeat composition among the six *Hydrangea* genotypes and to identify species-specific repetitive sequences, comparative read clustering was performed. The number of reads per sample ranged from 0.77 to 2.14 million (a total of 9 869 000 reads), corresponding to a genome coverage of 0.1× for the diploid genotypes and 0.05× for the assumed tetraploid *H. paniculata*. The RepeatExplorer2 pipeline reached an analysis limit by retrieving 8 492 761 reads. The total repeat proportion was calculated as 62.82 % (represented by 447 clusters within 302 superclusters, based on the minimum read number threshold of 0.01 % per cluster). Consequently, 37.18 % of the reads were considered non-repetitive.

**Fig. 5.**
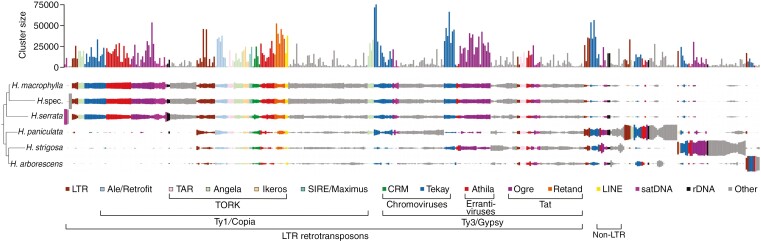
Comparative repeat composition among *Hydrangea* species. The bars represent the distribution of clusters comprising at least 850 reads (≥0.01 % of the analysed reads) among the analysed genotypes. Rectangles are coloured according to the type of repetitive element and their size is proportional to the genomic abundance in the respective genotype. The cladogram is based on the plastome-derived phylogeny (protein-coding and rRNA regions of the plastid genome; see Supplementary Data Fig. S2).

The six *Hydrangea* genotypes analysed were divided into four groups based on the composition and abundance of identified repeats in their genomes: Japanese diploid *Hydrangea* (*H. macrophylla*, *H. serrata* and *H.* spec.), Japanese polyploid *Hydrangea* (*H. paniculata*), Chinese *Hydrangea* (*H. strigosa*) and American *Hydrangea* (*H. arborescens*; Fig. 5). Whereas *H. macrophylla*, *H. serrata* and *H.* spec. share nearly all clusters, indicating a highly similar repeat composition among these three genotypes, the genomes of *H. paniculata*, *H. strigosa* and *H. arborescens* each show unique repeat patterns.

The genome of the polyploid *H. paniculat*a, which is endemic to Japan, has the largest overall repeat fraction among the analysed genotypes (>61 %; Table 2). Furthermore, it harbours several specific LTR retrotransposons, including Angela, Athila and Ogre members (one each) and three different Tekay elements. The similarly sized repeat fraction of the *H. strigosa* genome (>59 %; Table 2) includes two specific Ogre elements. The *H. arborescens* genome has the smallest total repeat fraction among the genotypes analysed (<43 %; Table 2), but also contains specific LTR retrotransposons, one of which is a Tekay element. Although the overall repeat composition of the *H. serrata* genome resembles those of *H. macrophylla* and *H.* spec., two *H. serrata*-specific satellite DNAs were identified within its genome.

### *Hallmarks of* Hydrangea *tandem repeats*

As tandem repeats often span large genomic regions, they are prime targets for the development of cytogenetic landmark probes. In addition, as they are among the most rapidly evolving genome components, they have potential as genome-specific probes. Hence, we focused on the most abundant *Hydrangea*-specific tandem repeats, delimiting their nucleotide sequence, abundance, degree of repetitiveness and their potential to serve as cytogenetic probes.

**Fig. 6.**
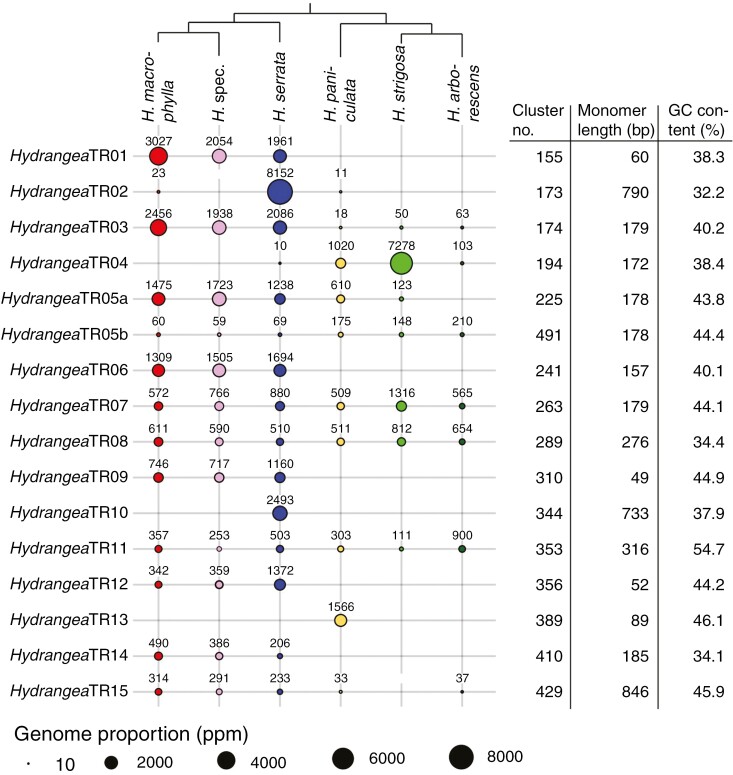
Quantification of satDNAs within different *Hydrangea* genomes and further *Hydrangea*TR characteristics. The relative satDNA abundance is indicated by the size of the circles, which corresponds to the genome proportion as parts per million (see key at bottom of figure). The cladogram is based on the plastome-derived phylogeny (protein-coding and rRNA regions of the plastid genome; see Supplementary Data Fig. S2).

The comparative clustering analysis revealed 16 different satDNAs with species-specific abundances among the six *Hydrangea* genotypes (Fig. 6). These satDNAs were named ‘*Hydrangea* tandem repeat’ (*Hydrangea*TR), and numbered based on their overall abundance throughout the six *Hydrangea* genomes. As two monomer variants were identified for *Hydrangea*TR05, the variants with the overall higher and lower abundances were called *Hydrangea*TR05a and *Hydrangea*TR05b, respectively. *Hydrangea*TR05a is abundant in the Japanese *Hydrangea* species including the polyploid *H. paniculata*. However, its variant *Hydrangea*TR05b is enriched in the *H. paniculata* as well as in the American *H. arborescens* genome (Fig. 6). Some *Hydrangea*TRs are enriched in certain *Hydrangea* species, such as *Hydrangea*TR04, which is comparatively abundant in the genomes of *H. paniculata* and *H. strigosa*. Species-specific satDNAs were identified for *H. serrata* (*Hydrangea*TR02 and 10) as well as for *H. paniculata* (*Hydrangea*TR13).

To determine whether the *Hydrangea*TRs have the capacity to form long tandem arrays and thus represent canonical satDNAs, we investigated their genomic environment on long reads. The monomer sequences of the 16 identified *Hydrangea*TRs were mapped to the long read dataset of *H. macrophylla* (GenBank accession number DRR231909; Nashima *et al*., 2021) and the continuity of the arrays was estimated visually using self-dotplots (Supplementary Data Fig. S4). Arrangement into continuous arrays could be verified for the 12 *Hydrangea*TRs that occur in the *H. macrophylla* genome, with the remaining *Hydrangea*TRs being restricted to other *Hydrangea* species. Therefore, the detection of long tandem arrangements of *Hydrangea*TR02, 04, 10 and 13 was not possible in *H. macrophylla* (Supplementary Data Fig. S4).

### Chromosomal localization of tandem repeats along hortensia chromosomes

**Fig. 7.**
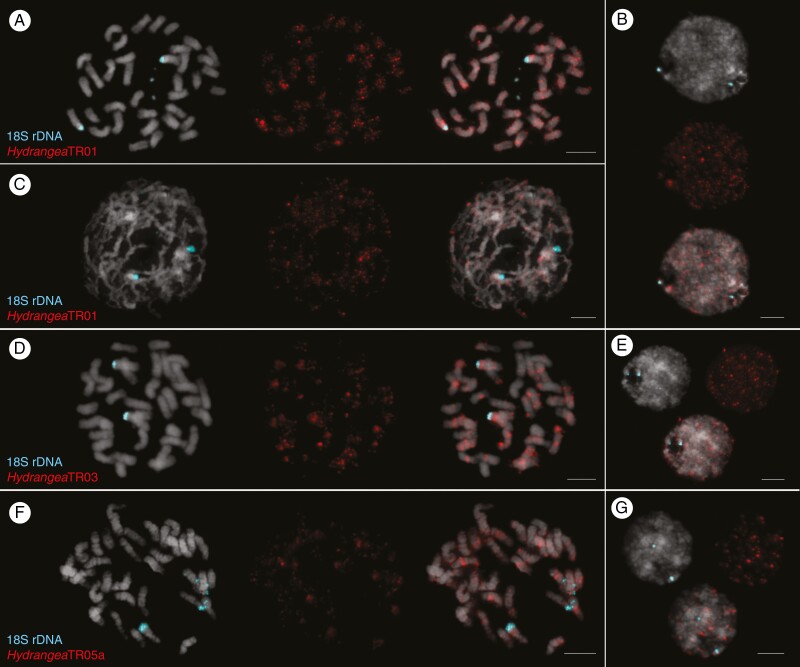
Multicolour FISH to chromosome spreads of *H. macrophylla* to determine the chromosomal localization of *Hydrangea*TR01, 03 and 05a. DAPI-stained mitotic chromosomes and interphase nuclei are shown in grey. Turquoise signals indicate the 18S rDNA loci. Metaphase (A, D, F) and prophase (C) nuclei display the dispersed occurrence of *Hydrangea*TR01, 03, and 05a along all 36 *H. macrophylla* chromosomes. Interphase nuclei of *H. macrophylla* show that *Hydrangea*TR01, 03 and 05a (B, E and G; red signals) are located in constitutive heterochromatic regions. Information on probe labelling and detection can be found in the Materials and methods section. Scale bars = 5 µm.

Based on the initial characterization of *Hydrangea* satDNAs, we selected the three most abundant tandem repeats of the *H. macrophylla* genome for FISH: *Hydrangea*TR01, 03 and 05a. To determine the chromosomal localization of these satDNAs, mitotic chromosomes of *H. macrophylla* (2*n* = 2*x* = 36) were hybridized with biotin-labelled probes marking *Hydrangea*TR01, 03 and 05a, respectively (Fig. 7, red signals), together with a probe marking the 18S rDNA as part of the 35S rDNA (Fig. 7, turquoise signals).

The two 18S rDNA signals in each mitotic nucleus indicate one chromosome pair (Fig. 7A, C, D, F; turquoise signals). In Fig. 7A and F, one 18S rDNA locus each is still decondensed, as shown by a stretched signal, indicating a despiralized chromatin thread. In comparison, in interphase nuclei the 18S rDNA signals are very concise, indicating a compact state of the corresponding chromatin (Fig. 7B, E, G; turquoise signals).

The three *Hydrangea*TRs show very similar distribution patterns. All three are found on all 36 *H. macrophylla* chromosomes. Generally, the *Hydrangea*TR signals are rather dispersed along the chromosomes with no clear site preference (both intercalary and distal positions). However, all three *Hydrangea*TRs are enriched on the chromosome arms containing the 18S rDNA. Furthermore, strong *Hydrangea*TR signals were often found near the centromeres, potentially contributing to the (peri)centromeric regions of these chromosomes (Fig. 7A, D, F). The dispersed distribution pattern of *Hydrangea*TR01 arrays is particularly visible in the prophase nucleus in which *Hydrangea*TR01 signals can be found all along the chromatin threads (Fig. 7C; red signals). As for *Hydrangea*TR01 and 03, most chromosomes harbour several strong signals, indicating major *Hydrangea*TR sites with presumably longer arrays, whereas the minority of chromosomes show only few, faint signals (Fig. 7A, D; red signals). The opposite applies for *Hydrangea*TR05a: most chromosomes harbour only a few, faint signals, indicating shorter *Hydrangea*TR05a arrays, whereas the minority of chromosomes show several strong signals, indicating longer arrays (Fig. 7F; red signals). Interphase nuclei demonstrate that the *Hydrangea*TRs are preferentially located in the DAPI-positive heterochromatic regions (Fig. 7B, E, G).

## DISCUSSION

To better understand the basis of the genomic variability among the hortensias, we chose a genotype panel of five *Hydrangea* species that covers a wide spectrum of different geographic origins, morphologies, base chromosome numbers and ploidy levels.

This panel serves as a basis for analysing genomic changes over evolutionary time scales and has the potential to guide hortensia breeding with respect to integration of wild germplasm resources. To develop a robust and useful dataset, we identify (1) plastomes for the evolutionary framework, (2) genome sizes and repeatomes to understand the overall genomic variation, and (3) chromosomal positions of selected repeat families.

### A phylogenetic framework for the six-hortensia panel

Despite the many efforts to elucidate comprehensive phylogenies within the genus *Hydrangea* (e.g. Samain *et al.*, 2010; De Smet *et al.*, 2015, 2017; Fu *et al.*, 2019; Raman *et al.*, 2023; Yang *et al.*, 2024), this is impeded by the vast diversity of *Hydrangea* species, subspecies and varieties. Therefore, we used plastome data to reconstruct a phylogenetic hypothesis, reflecting the relationship of the precise genotypes used in this study. This phylogeny is in line with other current phylogenies (i.e. Raman *et al*., 2023), dividing the analysed genotypes into two clades, one of them comprising the Japanese diploid species (including the undesignated *H.* spec.) and the other one comprising the polyploid and non-Japanese species. Depending on the fraction of sequence data (e.g. protein-coding versus full plastomes) and algorithm, incongruences became visible only in the relation of the three *Macrophyllae* members (*H. macrophylla*, *H. serrata* and *H.* spec.) to each other, which is due to the high degree of similarity of their plastomes and thus very short branch lengths.

### Hydrangea *genome sizes vary with chromosomal setup changes and repeatome dynamics*

Genome size estimation of two Japanese diploid *Hydrangea* species demonstrated that *H. macrophylla* (2127 ± 273 Mbp/1C) has a considerably larger genome than *H. serrata* (1660 ± 108 Mbp/1C). This was somewhat unexpected given their close relationship and the fact that both species have the same number of chromosomes (Cerbah *et al*., 2001). Indeed, Zonneveld *et al*. (2005) measured a genome size for *H. serrata* (2102 Mbp/1C) that is much more similar to that of *H. macrophylla* than the present estimation indicates. Thus, the question arises where such a difference may come from. It is unlikely that this observation is due to methodological inaccuracies since the genome size estimation for all hortensia genotypes analysed here was carried out with the same flow cytometer under the same conditions and even multiple measurements resulted in the same estimates. Hence, another possible explanation may apply: genome sizes can vary among individuals of the same species and can be associated with various environmental conditions of the respective regions of origin, such as altitude, temperature, light, humidity and soil (Bancheva and Greilhuber, 2006; Chalup *et al*., 2014; Inceer *et al*., 2018; Benhizia *et al*., 2021). The region where *H. macrophylla* grows is characterized by milder temperature seasonality in comparison with the more severe temperature changes in the snowy northern areas of Japan where *H. serrata* is growing (Iwatsuki *et al*., 2001). Additionally, the repeat fraction within the genomes may contribute to genome size differences as well, since we found more repetitive elements within the larger genome of *H. macrophylla* compared with that of *H. serrata*. The repeats, however, may be affected by the named environmental factors as well. In general, environmental stresses can impact repeat landscapes in plants. Some LTR retrotransposons are responsive to environmental stresses, such as heat, which can activate them and lead to increased transposition activity. This can result in increased copy numbers of retrotransposons and consequent increase in genome size (Papolu *et al*., 2022). In *H. serrata*, *H. strigosa* and *H. arborescens*, both our current findings and those from previous studies have demonstrated significant variability in genome sizes within the same species (Cerbah *et al.*, 2001; Zonneveld *et al*., 2005), indicating a high intraspecific variation in genome sizes among hortensias. It may be worthwhile to analyse further *H. serrata* genotypes in particular to explore the range in genome size span across *H. serrata* individuals.

The largest genome size among the analysed genotypes was measured for *H. paniculata* (3484 ± 52 Mbp/1C), which is a polyploid species (2*n* = 4*x* = 72 or 2*n* = 6*x* = 108; Funamoto and Tanaka, 1988). Its genome size is about twice as large as that of the diploid *Hydrangea* species, suggesting that the *H. paniculata* variety used in this study may be tetraploid. However, apart from the ploidy, the high repeat content (the highest among the analysed hortensias) also increases the genome size of *H. paniculata*.

Most *H. strigosa* accessions have 2*n* = 34 chromosomes; however, there are also a few individuals with 36 chromosomes (Mortreau *et al*., 2010). The relatively small genome size observed for *H. strigosa* (1428 Mbp/1C) may therefore be related to the loss of two chromosomes including considerable parts of the genome. This assumption is supported by the fact that for *H. strigosa* accessions having 36 chromosomes, larger genome sizes were estimated in comparison with the dysploid accessions (Mortreau *et al*., 2010).

The genome size of *H. arborescens* (770 Mbp/1C) estimated in this study is the smallest genome size measured for an *H. arborescens* accession so far (Plant DNA C-values Database). More comprehensive analyses focusing on *H. arborescens* intraspecies diversity are required to determine whether the reduced genome size of the *H. arborescens* variety ‘Annabelle’ may have arisen during the breeding process from the wild *H. arborescens* ancestor.

### *LTR retrotransposon composition may serve to distinguish* Hydrangea *genomes*

In comparison with other plant genomes (e.g. 73% in *Quinoa* and 68% in *Larix*; Heitkam *et al*., 2020, 2021) the analysed *Hydrangea* genomes show rather moderate overall repeat proportions (42.15–61.07%). However, given that diverged repeats escape the clustering threshold, the actual repeat content is likely to be higher. Repetitive elements are most abundant in the polyploid *H. paniculata* genome, whereas the *H. arborescens* genome is composed of the fewest repetitive elements (Table 2; Fig. 3; Supplementary Data Fig. S3), highlighting the correlation between repeat content and genome size that was observed for hortensias, similar to several other plants (e.g. Fabeae, *Solanum* and *Beta* sp.; Macas *et al*., 2015; Gaiero *et al*., 2019; Schmidt *et al*., 2023).

The repeat composition differs among the six *Hydrangea* representatives, dividing the Japanese diploids (*H. macrophylla* and *H. serrata*) and *H.* spec. from the polyploid *H. paniculata*, Chinese *H. strigosa* and American *H. arborescens*, which is well in line with the plastome-based phylogeny of these six genotypes.

In addition to the six hortensia genotypes from this study, the repeatome of the *H. macrophylla* cultivar ‘Sir Joseph Banks’ (GenBank accession number PRJEB32928) was analysed as well, since this is likely the first hortensia that was brought to Europe (Tränker *et al*., 2019). We found that its repeat profile is almost identical to that from the Japanese cultivar *H. macrophylla* f. *normalis* used in this study (Supplementary Data Fig. S5). Therefore, and due to the fact that the *H. macrophylla* cultivar ‘Sir Joseph Banks’ is considered to be the ancestor of the entirety of European hortensia breeding (Tränker *et al*., 2019), the following conclusions drawn based on the *H. macrophylla* f. *normalis* repeatome may also apply to most European *H. macrophylla* cultivars.

The undesignated *H.* spec. also shares nearly all repeat clusters with the two Japanese diploid *Hydrangea* species (*H. macrophylla* and *H. serrata*), including a similar respective repeat abundance. In combination with the plastome-based phylogeny that groups *H.* spec. closely with *H. macrophylla* and *H. serrata* (Supplementary Data Fig. S2), this indicates that *H.* spec. is a member of the section *Macrophyllae*. Thus, repeat profiles may be employed to ascertain the conspecificity of undesignated varieties. Interestingly, two repeats specific to *H.* spec. were observed, indicating minor differences in the repeat profiles among the *H. macrophylla* representatives that may be used to distinguish even closely related species or varieties (Fig. 5). Further analysis of these specific repeats may help to infer phylogenetic trajectories and to trace hybridization events (during cultivation). In other words, further repeatome analysis may assist in classifying unknown varieties within these species.

As in most plant genomes (reviewed by Weiss-Schneeweiss *et al*., 2015; Orozco-Arias *et al*., 2019), LTR retrotransposons are the most abundant repeats in *Hydrangea* genomes, accounting for 31–51 %. Members of the Ty3/Gypsy superfamily, especially Tekay elements, are the predominant repeats among them. In terms of genome share, the polyploid *H. paniculata* genome contains approximately four times more Tekay elements (963 Mbp/1C) than the diploid Japanese species (218–282 Mbp/1C) (Fig. 4, right). This increased abundance may indicate the acquisition of new Tekay elements as well as an amplificational burst of already present ones after the polyploidization event, as demonstrated by the comparative repeatome analysis.

In contrast, Ogre and Athila elements are less abundant in the polyploid *H. paniculata* genome compared with the Asian diploid species (*H. macrophylla*, *H. serrata* and *H. strigosa*). A possible explanation for this could be the efficient elimination of these elements from the *H. paniculata* genome or an increased Ogre/Athila amplification within as well as the acquisition of new Ogre/Athila elements into the Asian diploid *Hydrangea* genomes. Indeed, we detected a number of clusters annotated as Ogre and Athila retrotransposons, that specifically appear in the diploid *Hydrangea* genomes, indicating that integration events of Ogre and Athila sequences occurred several times after the split of the Asian *Hydrangea* species from their common ancestor.

Concluding, based on the pattern of shared repeat clusters among the analysed *Hydrangea* representatives as well as the assumption of a parsimonious evolution, the following scenario may be conceivable. The genome of the *Hydrangea* ancestor comprised a limited set of repeats. The geographic separation of the American *H. arborescens* from the remaining *Hydrangea* species led to the acquisition of distinct repeats, including some Tekay and Athila elements. The ancestor of the Asian branch acquired distinct repeats as well, including some Ogre and Retand elements. The subsequent split of the Chinese *H. strigosa* was followed by the acquisition of further Ogre and Athila elements. Within the Japanese branch, a polyploidization event resulted in the split of the polyploid *H. paniculata* from the remaining diploids. And whereas *H. paniculata* only acquired a few new repeats, the ancestor of the remaining diploids acquired a whole range of new Ogre and Athila elements (among others). Following this premise, the detection of Retand elements may be used to distinguish Asian from American *Hydrangea* species. A separation of Asian *Hydrangea* species based on Ty3/Gypsy retrotransposons may be possible as well. However, the specific retrotransposon families should be further characterized for this purpose.

The LTR retrotransposon composition of the Ty1/Copia superfamily shows similarly high interspecific variability among the *Hydrangea* genomes (Fig. 4, left). Whereas elements of the TORK lineage are the predominant Ty1/Copia representatives within the Asian diploid species (Angela elements within the Japanese diploid *Hydrangea* genomes, TAR elements within the Chinese *H. strigosa* genome), elements of the Retrofit lineage (Ale elements) are most abundant in the Japanese polyploid and the American species, indicating a high retrotransposon turnover during *Hydrangea* evolution. Such a fast evolution is typical for repetitive elements (Ugarkovic, 2005; Kuo *et al*., 2021). Species-specific Ty1/Copia retrotransposons were also identified (e.g. Oryco/Ivana elements within the *H. strigosa* genome), pointing to the integration of these elements into the *Hydrangea* genomes after *Hydrangea* speciation. Such interspecific differences in LTR retrotransposon composition have been reported in many flowering plant species, including differences on an intraspecific level (Du *et al*., 2010; Usai *et al*., 2017). However, no species-specific repeats were identified in the *H. macrophylla* genome, suggesting that this genome comprises the fundamental repeat set of Japanese diploid *Hydrangea* species, potentially resembling their common ancestor.

### *The two* Hydrangea *clades show differing satDNA profiles*

Conserved and specific satDNAs among the six hortensias were identified using comparative clustering analysis. The identification of satDNAs is a fundamental step in comparing the genetic diversity in plant species since satDNAs evolve fast and are therefore suitable as cytogenetic markers (Ugarkovic, 2005; Kuo *et al*., 2021).

The Chinese *H. strigosa* and American *H. arborescens* genomes are characterized by a lower satDNA variety and overall lower satDNA abundances compared with the Japanese *Hydrangea* genomes. Similar to the retrotransposon composition, the analysed *Macrophyllae* members (*H. macrophylla*, *H. serrata* and *H.* spec.) share most of their satDNAs. The appearance of genome-specific satDNAs (e.g. *Hydrangea*TR02 and 10 in the *H. serrata* genome) indicates a fast satDNA evolution, especially since the split of the Japanese diploid *Hydrangea* species (including *H. serrata*) occurred rather recently (within the range of 0.58–2.53 million years ago; Raman *et al*., 2023). Similarly, the species-specific satDNA amplification and the emergence of new monomer variants (e.g. *Hydrangea*TR05b) are hallmarks of the evolutionary drive (Fig. 6).

The fact that the *H. serrata*-specific satDNAs *Hydrangea*TR02 and 10 were not detected in the genome of the undesignated *H.* spec. (Fig. 6) gives rise to the assumption that *H.* spec. represents another *H. macrophylla* variety. Thus, the *H. serrata*-specific satDNAs *Hydrangea*TR02 and 10 may help to assign undesignated *Macrophyllae* species, even though the members of this section are genetically very close (Raman *et al*., 2023) and cannot even be resolved using an otherwise very robust plastome-based phylogeny (Supplementary Data Fig. S2).

### *Major tandem repeats in* Hydrangea *are distributed throughout the chromosomes*

A major 18S rDNA locus was detected on a pair of *H. macrophylla* chromosomes forming the secondary constriction site (Fig. 7). This observation is consistent with previous studies (Van Laere *et al*., 2008). The three most abundant tandem repeats in the *H. macrophylla* genome, *Hydrangea* TR01, 03 and 05a, were dispersed throughout the *H. macrophylla* chromosomes. Their distribution patterns are very similar. *Hydrangea*TR01 and 03 showed a high abundance on most chromosomes, whereas most *Hydrangea*TR05a chromosomes harbour only a few, marginal signals, indicating a low abundance. These signal intensities corroborate the bioinformatics results regarding the respective satDNA abundances.

Highly repeated distribution patterns at specific chromosome regions suggest that *Hydrangea*TRs may be involved in the maintenance of the heterochromatic structure, which is commonly observed in eukaryotic genomes including *A. thaliana* and rice (Garrido-Ramos, 2015). Large arrays of tandemly repeated sequences found in (peri)centromeric regions have also been detected in a wide range of species (Heslop-Harrison and Schwarzacher, 2011). All three TR signals were co-localized with 18S rDNA signals, suggesting that *Hydrangea*TRs are also interspersed in these ribosomal DNA arrays.

The presence of large as well as many small arrays on all chromosomes suggested that *Hydrangea*TRs in the *H. macrophylla* genome have not yet fully homogenized in the *H. macrophylla* genome, and may still undergo expansion, contraction and reorganization cycles. Transitions from transposable elements to satDNAs have been observed in some plant species, such as satDNAs in maize and *Solanum bulbocastanum* derived from LTR retrotransposons (Meštrović *et al*., 2015). SatDNAs and transposable elements are present in gene-poor heterochromatic regions, where the repeat copy number variation should not affect the genome stability. Therefore, *Hydrangea*TRs may have been generated as a result of their location adjacent to transposable elements in the *H. macrophylla* genome and may have accumulated at each chromosomal site by subsequent amplification.

Using bioinformatics (i.e. the RepeatExplorer2 pipeline), the three *Hydrangea*TRs were found in other *Hydrangea* species as well. The comparison with other Japanese diploid species may result in different signal patterns during FISH, i.e. if there are aberrations in the number of chromosomes. Other *Hydrangea*TRs that were found to be species-specific have the potential to be used as chromosome markers as well. Chromosome-specific satDNAs were identified and used for karyotyping the 34 chromosomes of *Crocus sativus* (Schmidt *et al*., 2019). Paesold *et al*. (2012) identified chromosome-specific markers for discriminating all nine chromosomes of *Beta vulgaris*, which were associated with centromeric, intercalary and subtelomeric regions of the chromosomes. Mortreau *et al*. (2010) reported the karyotyping of *H. involucrata* (2*n* = 30), and *H. strigosa* (2*n* = 34) by FISH using 5S and 18S-5.8S-26S rDNA and chromomycin A3 fluorochrome banding. The species/group-specificity and repeat abundance of *Hydrangea*TRs 02, 04, 10 and 13 make them promising candidates to serve as markers for further cytogenetic and phylogenetic analyses.

### Advancing breeding strategies using genetic markers and repeatome analysis

Using comparative low-coverage sequencing, our repeatome analysis provides insights into the genomic differences between hortensia species. We have identified repeat amplification and losses, as well as species-specific repetitive DNAs. Comparing the repeat abundance patterns with the plastome, it becomes clear that the repetitive DNAs carry a phylogenetic signal, as has been shown for other plant taxa (Dodsworth *et al*., 2015; Vitales *et al*., 2020; Herklotz *et al*., 2021). Presence/absence of repetitive DNA families or repeat polymorphisms can be used as a basis to develop molecular markers for the identification and differentiation of hortensia accessions, as done in barley and many other crops with the Inter-Retrotransposon Amplified Polymorphism (IRAP; Kalendar *et al.*, 1999), or in potato and poplar with the Inter-SINE Amplified Polymorphism (ISAP; Seibt *et al*., 2012; Reiche *et al.*, 2021). These markers can be helpful in the identification of potential parents and ancestors, and in the identification of important genes for specific traits. In addition, our repeat analyses may bring forward cytogenetic markers, as we have already explored here. In summary, plant breeders may benefit from the genetic information presented here to analyse inheritance patterns in progeny, hence, aiding in breeding, conservation and improvement of this ornamental species.

## Conclusions

Repeatome studies are essential for understanding plant genetic diversity. We performed a comparative repeatome analysis in *Hydrangea* using modern bioinformatic techniques and FISH-based chromosome mapping. Investigation of plastid genome polymorphism revealed genetic differences among *Hydrangea* lineages. The composition of tandem repeats suggested a closer relationship among Japanese diploid species. Further repeatome analysis may assist in classifying unknown *Hydrangea* varieties. The development of cytogenetic and molecular markers based on repeatome and plastome analyses is crucial for breeding and organizing genetic resources. Identifying specific sequences in Asian *H. macrophylla* and *H. serrata* and American *H. arborescens* can help manage world genetic resources. This comprehensive genomics analysis, including nuclear genome repeats, plastid and chromosome dynamics, is essential for future studies of *Hydrangea* genetic diversity.

## SUPPLEMENTARY DATA

Supplementary data are available at *Annals of Botany* online and consist of the following. Figure S1: circular plastid genome map of the six *Hydrangea* genotypes used in this study. Figure S2: plastome-based tree reconstructions of the studied *Hydrangea* genotypes using both RAxML (A, B, E, F, G, H) and IQ-TREE (C, D, I, J). Figure S3: repeat composition of the six *Hydrangea* genotypes. Figure S4: *Hydrangea*TR characteristics determined by RepeatExplorer2 and self-dotplot analyses. Figure S5: comparative repeat composition between two *H. macrophylla* cultivars, *H. macrophylla* f. *normalis* (used in this study) and *H. macrophylla* ‘Sir Joseph Banks’ (main basis for all *Hydrangea* breeding in Europe; Tränker *et al.*, 2019). Table S1: primer sequences for the amplification of different *Hydrangea*TRs. Table S2: read datasets used for repeatome analyses and the reconstruction of plastome sequences. Table S3: absolute amount (Mbp) of different repeat classes in six *Hydrangea* genotypes. Data S1: *Hydrangea*TR nucleotide sequences derived as RepeatExplorer2 consensuses from the comparative analysis. Data S2: *Hydrangea*TR sequences used as FISH probes.

mcae184_suppl_Supplementary_Material
