# Supplementary material for: Repeatome landscapes and cytogenetics of hortensias provide a framework to trace Hydrangea evolution and domestication
Source: Ann Bot. 2025 Jan 23;135(3):549–64. doi: 10.1093/aob/mcae184 (PMC11897596; doi:10.1093/aob/mcae184)
Supplement: mcae184_suppl_Supplementary_Material [file mcae184_suppl_supplementary_material.docx]

Repeatome landscapes and cytogenetics of hortensias provide a framework to trace *Hydrangea* evolution and domestication

**Sara Ishiguro^1+^, Shota Taniguchi^1+^, Nicola Schmidt^2,3+^, Matthias Jost^4,5^, Stefan Wanke^2,4,5,6^, Tony Heitkam^2,3^*****, Nobuko Ohmido^1^***

**^+^** Equally contribution as first authors

*equally correspondence

1 Graduate School of Human Development and Environment, Kobe University, Nada-ku, Kobe, 657-8501, Japan

2 Faculty of Biology, Technische Universität Dresden, D-01069 Dresden, Germany

3 Institute of Biology I, RWTH Aachen University, 52056 Aachen, Germany

4 Institut für Ökologie, Evolution und Diversität, Goethe-Universität Frankfurt, 60438 Frankfurt am Main, Germany

5 Departamento de Botánica, Instituto de Biología, Universidad Nacional Autónoma de México, Mexico City, Mexico

6 Abteilung Botanik und Molekulare Evolutionsforschung, Senckenberg Gesellschaft für Naturforschung, 60325 Frankfurt am Main, Germany

##

## Supplementary Data Figure S1


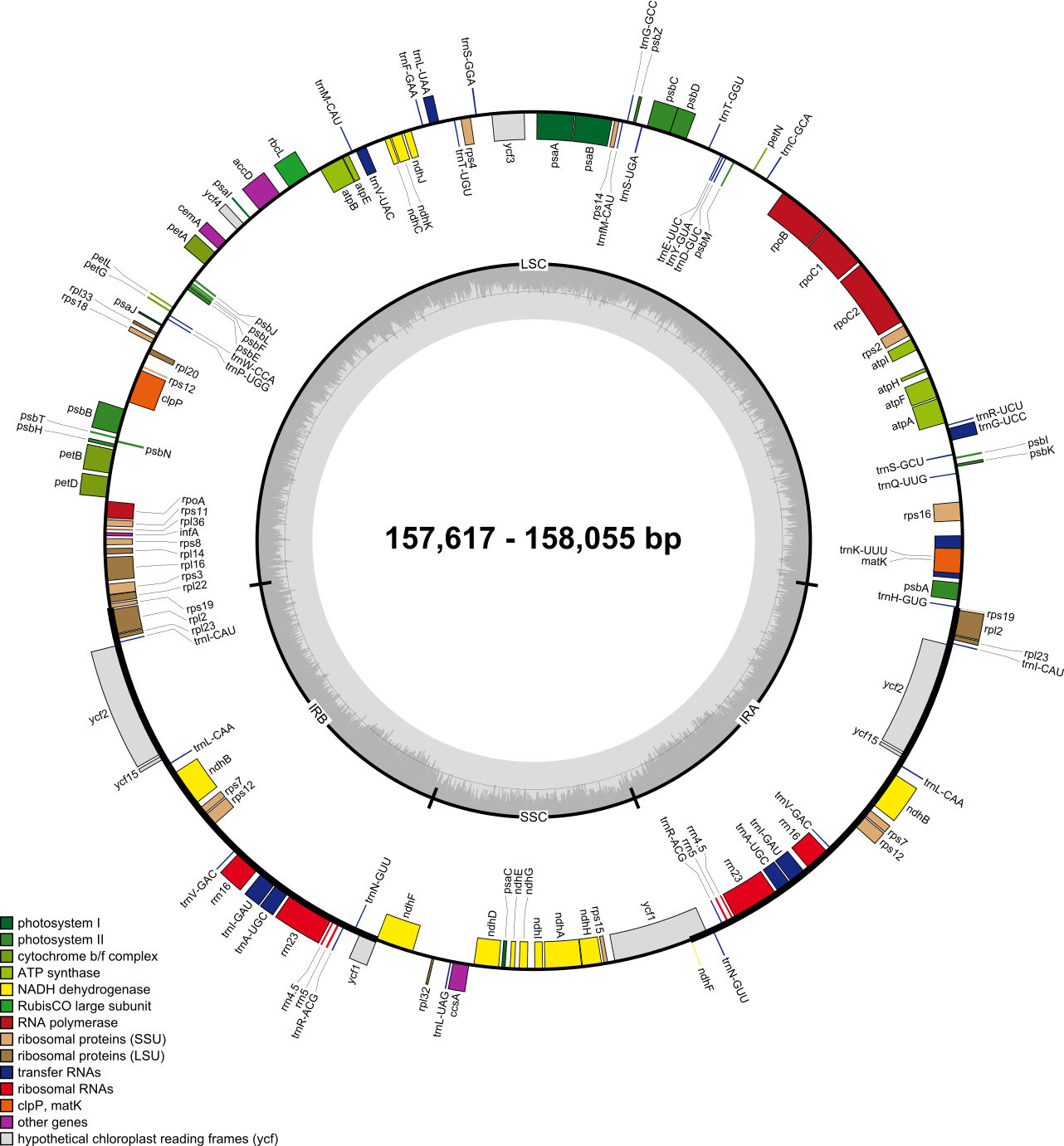


**Fig. S1: Circular plastid genome map of the six *Hydrangea* genotypes used in this study.** Different colors indicate genes of different functions. The genes inside the outer circle are transcribed clockwise and the genes on the outside are transcribed counter clockwise. The large single copy (LSC) region, small single copy (SSC) region, and two inverted repeat (IRA and IRB) copies are indicated on the inner circle. The gray circle visualized the GC content across the different plastome regions.

## Supplementary Data Figure S2


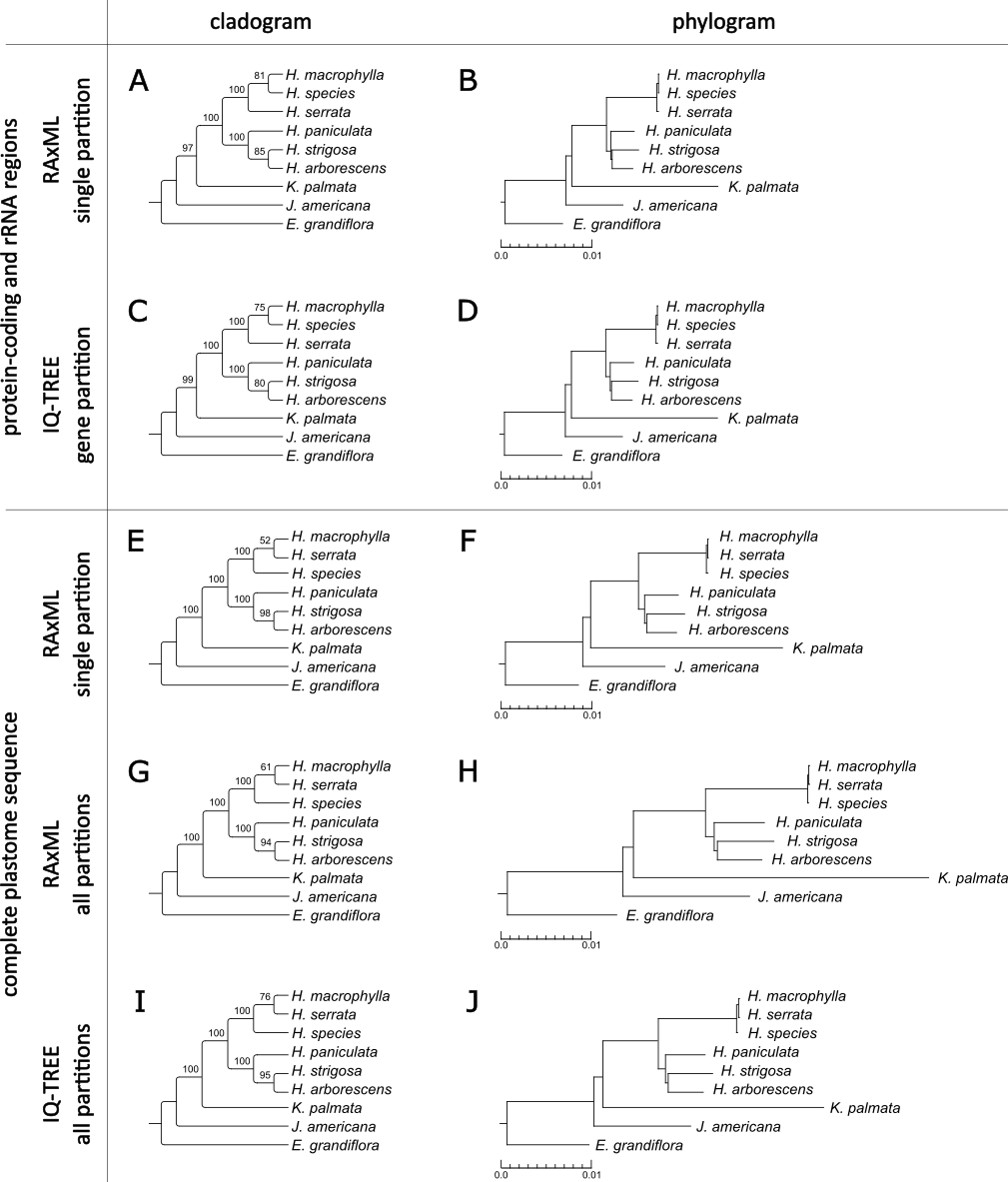


**Fig. S2: Plastome-based tree reconstructions of the studied *Hydrangea* genotypes using both RAxML (A, B, E, F, G, H) and IQ-TREE (C, D, I, J).** The topologies were calculated based on the protein-coding and rRNA regions (single partition: A, B; gene partition: C, D), as well as on the complete plastome sequence excluding one IR copy (single partition: E, F; all partitions [genes, introns, spacer]: G-J). On the left side (A, C, E, G, I), the cladograms highlighting the bootstrap values are depicted. Support values are based on 1,000 bootstrap replicates. On the right side (B, D, F, H, J), the corresponding phylograms, drawn to scale, are shown. The outgroup sampling is comprised of representatives from the sister tribe Philadelpheae (*Kirengeshoma palmata*: NC_044808) and the sister subfamily Jamesioideae (*Jamesia americana*: NC_044836). *Eucnide grandiflora* (NC_044767), a member of sister family Loasaceae, was used for rooting.

## Supplementary Data Figure S3


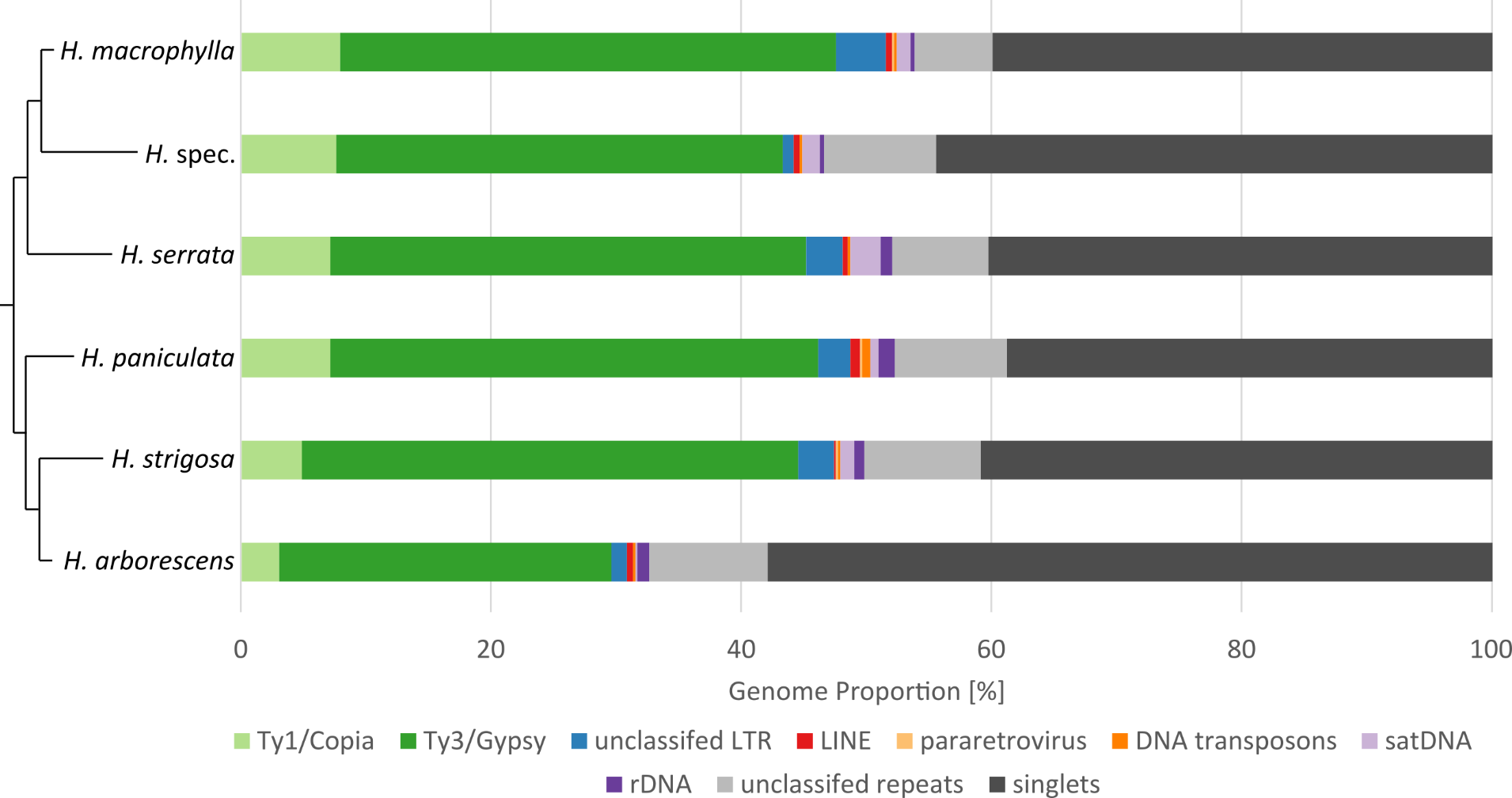


**Fig. S3: Repeat composition of the six *Hydrangea* genotypes.** The displayed cladogram is based on the plastome-derived phylogeny (protein-coding and rRNA regions of the plastid genome; see Supplemental Data Fig. S2). The composition of main repeat classes is shown as percentages of the respective genome.

## Supplementary Data Figure S4


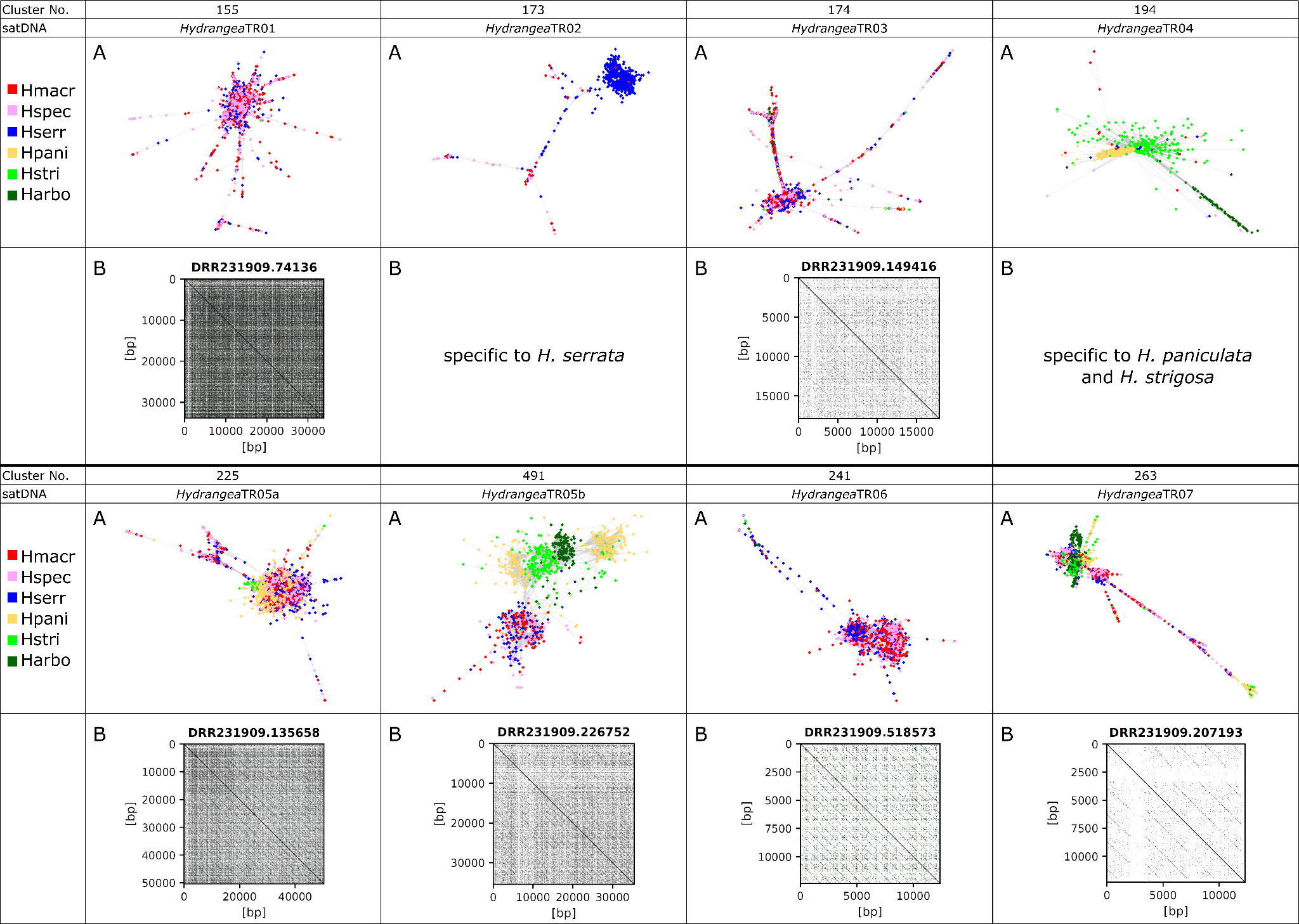


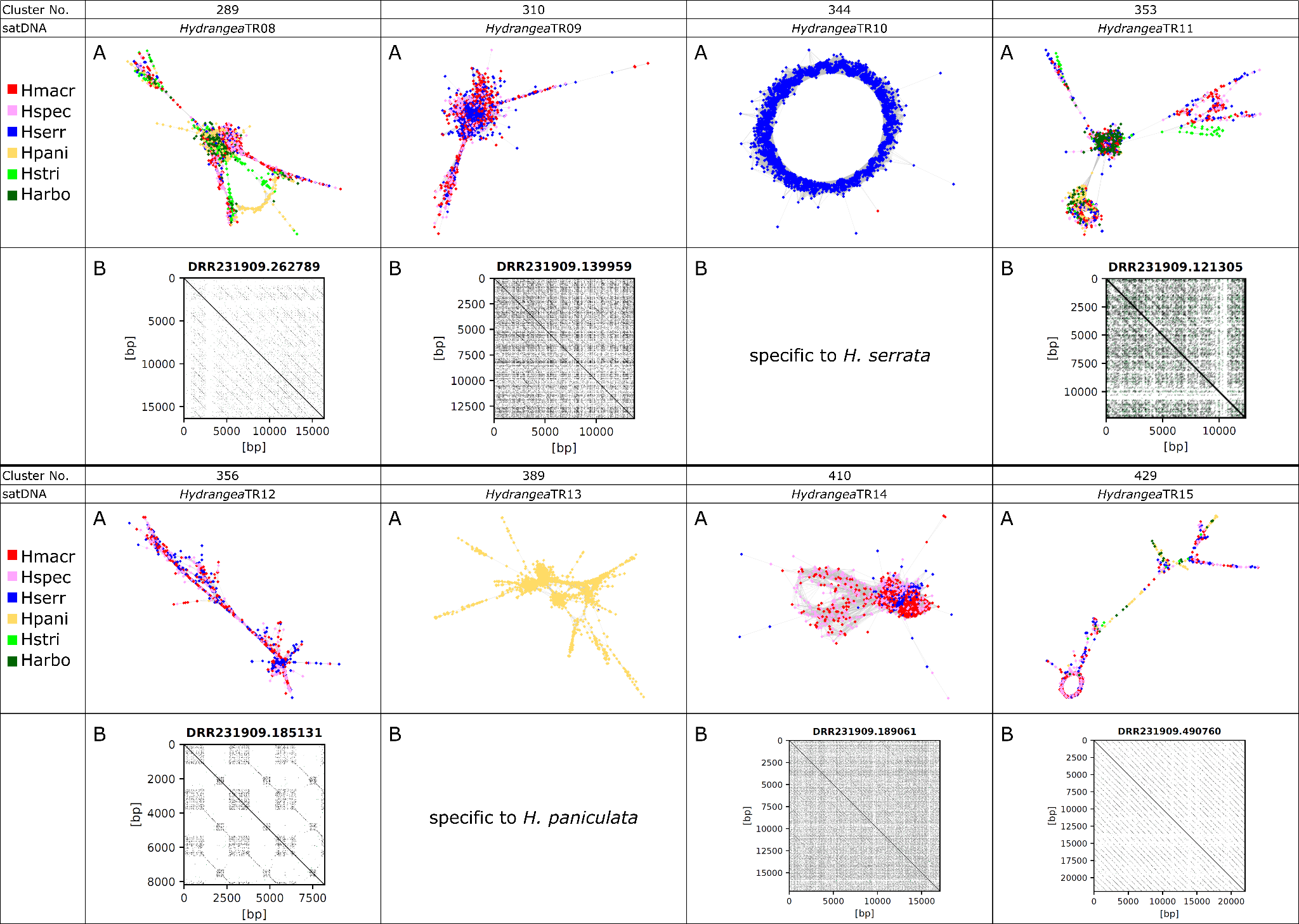


**Fig. S4: *Hydrangea*TR characteristics determined by RepeatExplorer2 and self-dotplot analyses.** (A) SatDNA-typical bulk or ring-shaped cluster graphs resulting from the comparative RE2 analysis of the six different *Hydrangea* genotypes. The origin of the distinct reads is color-coded and highlights *Hydrangea*TR specificities. (B) Arrangement of *Hydrangea*TR monomers on long reads of *H. macrophylla* (DRX222164). *Hydrangea*TRs that are specific to other *Hydrangea* species could not be detected in the *H. macrophylla* dataset. Forward matches are marked by black lines, whereas reverse matches are indicated in green. To account for the ONT read error rate of 5-10%, we chose a word size of 20 bp and allowed five mismatches. Abbreviations: Hmacr = *H. macrophylla*, Hspec = *H.* spec, Hserr = *H. serrata*, Hpani = *H. paniculata*, Hstri = *H. strigosa*, Harbo = *H. arborescens*.

## Supplementary Data Figure S5


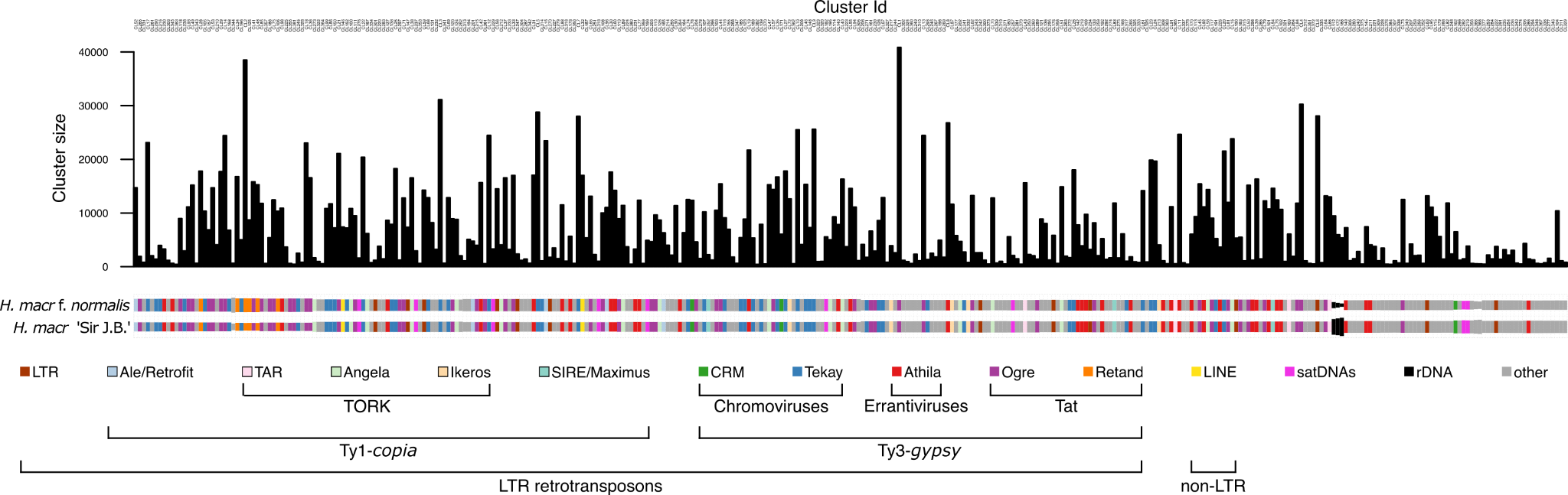


**Fig. S5: Comparative repeat composition between two *H. macrophylla* cultivars, *H. macrophylla* f. *normalis* (used in this study) and *H. macrophylla* ‘Sir Josef Banks’ (main basis for all *Hydrangea* breeding in Europe; Tränker *et al*., 2019).** The bars represent the distribution of clusters comprising at least 432 reads ( ≥ 0.01% of the analyzed reads) among the analyzed species. Rectangles are colored according to the type of repetitive element and their size is proportional to the genomic abundance in the respective cultivar.

## Supplementary Data Table S1

**Table S1: Primer sequences for the amplification of different *Hydrangea*TRs.** *Hydrangea*TR01, 03, and 05a were amplified using genomic DNA of *H. macrophylla*.

| **Primer name** | **FW Sequence** | **RV Sequence** | **Ann. Temp. [°C]** |
| --- | --- | --- | --- |
| *Hydrangea*TR01 | CGT TAT ATT CTC AAA ACG AGG G | CGG TTC TCG CAA CAG ATT TG | 55 |
| *Hydrangea*TR03 | CCG ATT CAC TAC TCA AAA CCT CA | AAC GCC ACG AAA CCT TAC AC | 55 |
| *Hydrangea*TR05a | TCA AAA TGC AAA CCG TCG AT | GAG TCC GAT TCC CGT TCC | 53 |

## Supplementary Data Table S2

**Table S2: Read datasets used for repeatome analyses and the reconstruction of plastome sequences.** The reads were sequenced using the Illumina Novaseq 6000 system as 150 bp paired-end reads for each genotype and published as part of the study ERP151402.

| **Genotype** | **Accession no./ENA Run ID**  **(Illumina reads)** | **Experiment**  **(Illumina reads)** | **Accession no. of the corresponding plastome sequence** |
| --- | --- | --- | --- |
| *H. macrophylla* | ERR12526782 | ERX11900996 | OR701877 |
| *H.* spec. | ERR12526783 | ERX11900997 | OR701880 |
| *H. serrata* | ERR12526784 | ERX11900998 | OR701879 |
| *H. paniculata* | ERR12526785 | ERX11900999 | OR701878 |
| *H. strigosa* | ERR12526786 | ERX11901000 | OR701881 |
| *H. arborescens* | ERR12526787 | ERX11901001 | OR701882 |

##

## Supplementary Data Table S3

**Table S2: Absolute amount [Mbp] of different repeat classes in six *Hydrangea* genotypes.** Abbreviations: Hmacr = *H. macrophylla*, Hserr = *H. serrata*, Hpani = *H. paniculata*, Hstri = *H. strigosa*, Harbo = *H. arborescens*, Hspec = *H.* spec.

| **Repeat** |  | **Lineage** | **Clade** | **Genome Fraction [Mbp]** | | | | |  |
| --- | --- | --- | --- | --- | --- | --- | --- | --- | --- |
|  |  |  |  | **Hmacr** | **Hserr** | **Hpani** | **Hstri** | **Harbo** | **Hspec** |
| **LTR retro-**  **trans-**  **posons** | **Ty1/Copia** | Retrofit/Ale |  | 30.63 | 26.29 | 84.66 | 17.65 | 8.09 | 31.63 |
|  |  | Alesia |  | 0.00 | 0.00 | 0.00 | 0.15 | 0.00 | 0.00 |
|  |  | Oryco/Ikeros |  | 15.74 | 11.02 | 16.37 | 7.97 | 2.70 | 14.58 |
|  |  | TORK/Tork |  | 2.13 | 1.99 | 23.34 | 8.18 | 1.08 | 1.33 |
|  |  | TORK/Angela |  | 92.74 | 63.82 | 83.27 | 7.02 | 8.86 | 70.46 |
|  |  | TORK/TAR |  | 15.53 | 8.92 | 20.90 | 16.97 | 2.54 | 14.58 |
|  |  | TORK/Ivana |  | 0.00 | 0.30 | 1.05 | 2.38 | 0.00 | 0.00 |
|  |  | SIRE |  | 13.61 | 6.89 | 12.19 | 5.15 | 0.85 | 11.55 |
|  |  | Bianca |  | 1.49 | 1.57 | 8.01 | 5.17 | 0.46 | 1.33 |
|  |  | **Total Ty1/Copia** |  | **171.86** | **120.81** | **249.80** | **70.65** | **24.56** | **145.46** |
|  | **Ty3/Gypsy** | chromovirus | CRM | 12.76 | 11.13 | 17.42 | 7.58 | 2.93 | 11.55 |
|  |  |  | Tekay | 282.68 | 218.18 | 963.33 | 251.52 | 148.23 | 223.87 |
|  |  | Non-chromovirus | Athila | 183.56 | 130.24 | 105.22 | 25.69 | 45.58 | 129.74 |
|  |  |  | Ogre | 295.23 | 213.17 | 169.67 | 233.64 | 4.08 | 249.82 |
|  |  |  | Retand | 65.30 | 56.20 | 101.04 | 45.16 | 2.16 | 58.71 |
|  |  | **Total Ty3/Gypsy** |  | **839.53** | **628.92** | **1356.67** | **563.60** | **202.97** | **673.70** |
|  | Unclass. LTR | | | 83.17 | 47.97 | 90.24 | 41.27 | 9.78 | 17.27 |
|  | **Total LTR** | |  | **1094.55** | **797.70** | **1696.71** | **675.52** | **237.31** | **836.39** |
| LINE |  |  |  | 11.06 | 7.80 | 24.39 | 3.43 | 4.85 | 8.33 |
| Pararetrovirus | | | | 2.98 | 0.00 | 6.62 | 1.14 | 0.00 | 0.00 |
|  | **Total retrotransposons** | |  | **1108.59** | **805.51** | **1727.72** | **680.09** | **242.17** | **844.72** |
| **DNA transposon** | TIR | EnSpm_CACTA |  | 0.43 | 0.00 | 3.83 | 0.00 | 0.08 | 0.38 |
|  |  | hAT |  | 1.91 | 0.67 | 8.71 | 0.56 | 0.15 | 2.84 |
|  |  | MuDR_Mutator |  | 2.77 | 0.94 | 9.06 | 1.81 | 0.31 | 2.27 |
|  |  | PIF_Harbinger |  | 0.00 | 0.00 | 3.14 | 0.00 | 0.00 | 0.00 |
|  | Helitron |  |  | 0.00 | 0.00 | 0.70 | 0.00 | 0.00 | 0.00 |
|  | **Total DNA transposons** | |  | **5.10** | **1.60** | **25.43** | **2.37** | **0.54** | **5.49** |
| **Tandem repeats** | satDNAs |  |  | 24.67 | 39.39 | 19.86 | 15.97 | 1.85 | 24.81 |
|  | rDNA | 35S |  | 4.89 | 16.37 | 47.03 | 9.71 | 7.01 | 7.77 |
|  |  | 5S |  | 0.00 | 1.59 | 0.35 | 1.53 | 0.23 | 0.00 |
| Unclass. repeats | | | | 134.00 | 126.46 | 307.29 | 133.12 | 72.69 | 168.76 |
| **Total repeats** | | | | **1276.84** | **990.85** | **2128.03** | **842.66** | **324.56** | **1051.55** |

##

##

##

## Supplementary Data S1:

**Data S1: *Hydrangea*TR nucleotide sequences derived as RepeatExplorer2 consensuses from the comparative analysis.** The start and the end of the monomer is arbitrary. Primer binding sites (corresponding to the primer sequences listed in Table S1) are accentuated by bold, underlined fonts.

>HydrangeaTR01-consensusmonomer_60bp

C**CGTTATATTCTCAAAACGAGGG**TTAACG**CAAATCTGTTGCGAGAACCG**AGAATATAAAT

>HydrangeaTR02-consensusmonomer_790bp

AAATATAGGTACAACATGCCATAGCATAGGAACAGTATGGTATAGCCAAGATATTGGATGAAAACAATACTCTTAAAGACTATTCCTGGAGATAGTCTCTTTGTCTACGTACACCAGGTTCCCGAATACAGACTCTTAAAGCAAATTCCAGGAGATAGTCTCTGATTATATTTGATTATCCATTCCAGAGACAAACTATGTCATTTTCTATTTTACATATTGGATACAAAGATACCTATTGTAAGACTTTTTTCATAGACAGTCTAGTTATGTGTCTTTGGCCAGACTGTATTTCTGTTAGCATATCTGCATTGTCCAGCGATAAAAAAAATACACAAACAGATAAACAACAAAATCACATACCTCTTATACCAAAATGATAACATATCAAAGAACTGAAAGTTGTAAGACTTTTGAGATGAGGACTTTGAGATTTGAAATTCAAAGTTCAGTAGTGAGAAACTTAAATTTTTGGACAAAACAGTTTTTGGTTCTTTTTAACATGATGATTTATGGGATGAAAATAGCACTTCCAAAGACTATTCCCGGAGATAGTCTCAATAAAACTCTTTCGAAAAACTATTCGCGGTGATATTCTCTTAGTTTTTGTACAACATGGCATAGTTTACATACATGATGATACATCAAATAATAGTATAAGTACAATGTGGTACAACCTTGTACTGCATGGCATGTTATACCGTAGTAGAGTGTAAGTATAACATGCTATGTCATATTATAGTGTAGGTATAGTGTGTTATAGTATAGGTTCAATATTTTTTTCAAAAAA

>HydrangeaTR03-consensusmonomer_179bp

TTA**CCGATTCACTACTCAAAACCTCA**AAAACTAAGCTTTTTCAAGCCTAAGTGACTAAAGAGTCATTTTTTCGCATTTTTTGTGAACCGTGAATCCGTTATGCATTCCGTTAGTTCCCGCAAGTTTATTTAGGCATGTTTGAGGTGC**GTGTAAGGTTTCGTGGCGTT**TGGTTAAGCGGT

>HydrangeaTR04-consensusmonomer_172bp

GTGTAAGTGCAAAACATAAATGTCGAGTTTTCACCCGACCCTCACACACAAGTTATAAAACACCAAAATACACGTGTTCAAATTGACTTTCTAGAAAAACTTGACTGGTGAGAGGTAGTTCGTGAGTTATTTGACCCGAAATGGCCTTTTCGGCAATTTTTAAAATTGGAGG

>HydrangeaTR05a-consensusmonomer_178bp

CACTACAAAACAACTTTTTTACCTCGTAGACTATGAAAAACTAAGTTTTTGGGGTTTGGGGGG**TCAAAATGCAAACCGTCGAT**CCGGTAACCACGAAACTTAACTCGCACCTCCCACACGTCTAAAGAAAGTTGTAGGAAGAAAC**GGAACGGGAATCGGACTC**CCGGTTATCAAGATA

>HydrangeaTR05b-consensusmonomer_178bp

CGCGAAAAAATAGAATTTCAAACACTTAGGCTTTAGAAAACTTAGTTTTGGGGTTTTGACGGGTGAAACGGTGAACCGCCAATCGGAATGACACGAAACTTGACCGACACCTCACACACGCCTAAAGAAAGTTGTAGGAACAAACGGGACCCTTATCGGATTCCCGGTTTGAGAGTTA

>HydrangeaTR06-consensusmonomer_157bp

GGTATAATTAACAAATATGGGGAATAATGAACAAATATGCAACATGGGAGATGACAACTATTGTAGGGGAATCCCCCACAATTGTAAAGAATCCCCCACAGTTTGTCTAGAGTCCCCCACAAACTGTTGTATATGGGGGATTCTTAACAAACTGTGG

>HydrangeaTR07-consensusmonomer_179bp

TCTCTCAAAACCTCAAAACTTAGTTTTAGAAGGCCTAAGTCTATGAATTTGCGTATTTGCGTCTAACTTGCAATCCGTGAGTCGGAATTGCGTGCCGTTTCTTCCTACAAGTCTATTTAGGTGAGTATGAGGTGTAGGTTCCAGTCTCGTGTCATTCCGAGTGCCGGAACCCATTTTGG

>HydrangeaTR08-consensusmonomer_276bp

TGGGGCTTACATTTAATAAGTTAATATAATTAAAGTCTTCAAATAATACGGAGTGACCTACCCCAAGGTTTTTCGACGTTCTGGATCCAATGGTAGGGTCCATTTGGCCTGAATATATTTGTAAAATGACTATTTTGCCCTTGAAACTTTAAAATGCCATAACTTGTTAAGATTAAACCAGAATTGAACGTCGTCAGAGCCTGTGAGCTCATATTAATATGAAGAACATTTTGAGACCTAAAAAGTACATATATCGTAAAATGACCAAATTGCCCT

>HydrangeaTR09-consensusmonomer_49bp

TATCGTGAGCTTCCTCACTCCTTTGTCCAAAATCGTGAGCTTTCTCACT

>HydrangeaTR10-consensusmonomer_733bp

TTTGATACTCCATTGACTGTTAGTTGAGTACTATGGGGGGAACACGCCAATCGTTTTTGGACCCTTGTCACCTCTCCATCTATGTGCACCAACTAATTCTGGAATATAAATAATAAATATACACCCGCCCCCCAGTCACATACAGTCAAAAATGCTAAATGATTATTCATCTGATTACTCAACAGCCCTCAGATCTCTCTATAACTTGAGAGTGTTTTACCTAAAAAAAAGTCACATACAGAGAGTGATTTTTGAGGGGTTTCTCTAACATAAAATATGCCTCCACAATCCTCAGGTATTGTTCTTGTTCTTCCAGTGATCTATTTCTTCGATCTTCTTCTTGCTAATTTTTTGTTCCCCTTTTCTCATCTTTGATATATAAAATACTACCCAAATAACCAAATAGATACCCAAATTTAATTACTAGAGTTGCTCTAAGGAAGCATTTAATGCTTGGCGTGAGGGAGTTGGCAAAAGATATCACGTGAAAGGTGGTTTGACAATTTGGGGCGCTAGAAACATGCTGAGTACTGGCTACTCTCATTAATGCTGTCACCCCTTTCTTTTGGGAAAAGATATCACGTGATTCCCCTATAATCTGTGTTTTTATAATTCCAGAATTTAAATGACCGCGTTTGGGAAGATGTGGGCAATAATGGGTGCGTGGACTATAGTTCTCCATCAACAAACACATTGGCTAATTTGGCTGGCTTTCTAGCCAAAAAAAAATAAA

>HydrangeaTR11-consensusmonomer_316bp

ACGACCCTGGTGTTGTGGGCGGGCTGCTGTAGGTGCCTTTGTGGCTGCAGTAAGTGCTGGCAGGCTACACACTATGGGAAGTTCGTCCCACATCGCCTGGGTGTGGAAAAGTAATGTGCTATATATGTGTGGTTCCAAGTCTCCCTAGTAAGAGGCCTTTTGGGTAGTGGCCCAAGAACAAATCCGTGCGGGTTTGGGCCCAAAGCGGACAATATCTTACTGAGCGAGACTTGGGTCGTGACAGAATGGTATCAGAGCTAGACCCAGCCGGAAGTGTGCCAACGCGGACGTTGGGCCCTAAGGGGGGTGGAATGTA

>HydrangeaTR12-consensusmonomer_52bp

TCCTCGGTCTTGGTATAAGTCGTCTATCGCATCCTATGAGTTACCGATCAAA

>HydrangeaTR13-consensusmonomer_89bp

CTTGGAACATAAGGCGCCAAGGGGTGCAGTTCATTTCTGGTTTAGCATTGGAGCCTAAGGCATTAAGACGCAGATCATTGCTATTGTTG

>HydrangeaTR14-consensusmonomer_185bp

GGAGGACGGTACAAATGCTCGTGTTCGGATTACGGTAAGACTAAAATAAACATTTGAAATTGGACTGTAAAACAAATTGAGATATCGAAAGAACTTTCCTTGTAAAGACGGAAATTATGAGTACCACATATGGAAATAAATTCCCACCAGTGGGATTTTTTTGAGTCTAAAATTGCAATTTTGTT

>HydrangeaTR15-consensusmonomer_846bp

CTAAGCTAGGTTCTGGCGCCGAGAACCTTCATGCACAGACTCACGTAGGCTCTGCAGGCAGTCACATGGTTTGTGATGCTTTTACCCGAGAATCCGGAAACTTGTCTCGGCATGACCCTAAGAGCATGATGCAAGTTACAAATATCCAAGGTAGTGCTTGCCACGTGGCAACAACCTCACCAAATGTTCAAATGCCAACCACTTTGTCATTACATGACCCTGCTGGCCATCTATTTACGCATACCTGCTTGAGTGGTAATGAGGATGTTTGTACCACTAATCAAGAGAATGCTACCACCCCATCACAGCACATAACCACGGCTCCTATTCCTAAGGCTGGAGTTGTTACAAAAGGTTCTTGCACAGCCCCACACCAGTCTGATACCCAAGTGAACCCTACCTACTGTGAGGCACTGAAGGCTAAATTACATAGTGATAACTCTGCTACGGCTAACTCCTCTGCAAAGCACATCCAAGAGACAAGCTGGGTTTCCCTATTGTAACCTAAAATAGCTACAGAGTTTAAAGTCCATTATATCAAGCACCAGAGAGATATGGCCAGAATTCAAATTAGGGTGCCTAGGATATTGACAGATCAAGGCAGCAAAGCTTGGGAGTACACTCTTGTGGGGCACTTCATAGGTAAGAAGCTTCCCTACTCTTTGGTGAAATCTGCATCCTCTAGACTCTGGGGTAGATTGGGACTTGAAGATATGTTAGCTATGGAATCAGGCTACTTCTTCTTCAAATTCAATTCCCAAGATGAAAGTGCTGCCTCATGTTTTCCAGCTGGGAAAAGATCTGCAGCTGATCAGAACTTAGTTCTTGGCTTGACGGCTTGCTCAG

## Supplementary Data S2:

**Data S2: *Hydrangea*TR sequences used as FISH probes.** The alternating monomers are indicated by a change in color. Light markings indicate partial monomers.

>HydrangeaTR01-Hmac_207bp_OY742023.1

TCGTTATATTCTCAAAACGAGGGTTAACGCAAATCTGTTGCGAGAACCGAGAATATAAATCTGTTATATTCCAAAACGGGGGTTAACGCAAATATGTTGCGAGAACCGAGAATATAAATCTGTTATATTCCAAAACGGGGGTTAACGCAAATCTGTTGCGAGAACCGAGAACATAAATCTGTTATATTCCAAAAAGGGGGGTTAAAT

>HydrangeaTR03-Hmac_580bp_OY742025.1

GTATGTTTGAGGAGCGTGTAAGGTTTCGTGGCGTTTGGATAAGCGGTTTACCGATTAACTCCTCAAAACCTCAATACTTAAGCTTTTTCAAGCATAAGTGTCTAAAGAGGCACTTTTTCGCATTTTTTCGCATTTTTTTTCAGCCGTGAATCCGTAAAGCATTCCGTTAGTTCCCACAAGTTTATTTAGGTATGTTTGAGGTGCGTGTAAGGTTTCGTGGCGTTTGGTTAAGCGGTTTACCGATTAACTGCTCAAAACCTCAATACTTAAGCTTTTTCAAGCATAAGTGTCTAAAGAGCCACTTTTTCGCATTTTTTTTGAACCGTGAATCCGTAAAGGATTCCGTTAGTTTACGCAAGTTTATGTAGGTATGTTTGAGGTGCGTGTAAGGTTTCGTGGCATTTGGTTAAGCGGTTTACCGATTCACTACTCAAAACCTCAATACTTAAGCTTTTTCAAGCATAAGTGTCTAAAGAGGCACTTTTTCGCATTTTTTTTGAACCGTGAATCCGTAAAGCATTCCGTTAGTTCCCGCAAGTTTATTTAGGTATGTTAGAGGTGCGTGTAAGGTTTCGTGGCG

>HydrangeaTR05a-Hmac_448bp_OY742027.1

CAAACCGTCGATCCGGTAACCACGAAACTTAATTCGCACCTCCCACACGTCCATAGAAAGTAATAAGAAGAAACAGAACAGGAATTGGACTCCCGGTTACCAAGATACAATATAAATCAACTTTTTTACCTCATAAACTCTTAAAAACTAAGTTTTTGGGGTTTGGGGGGTCAAAATTCAAACCGTCGATTCGGTAACCACGAAACATAACTCGTGCTTCCCACACGTCCATAGAAAGTTGTAGGAAGAAACGGAACAGGAATTGGACTCCCGGTAATCAAGATACAATACAAAACAACTTTTTCACCTTATAGACTATGAAAAATTAAGTTTTTGGGGTTTCGGAGATCAAAATGCAAACCGTCGATCTGGTAACCACGAAACTTAAGTCTCACCTCCCACAAATATAAAGAAAGTTGTAGGAAGAAACGGAACGGGAATCGGACTC
